# Challenges and opportunities when moving food production and consumption toward sustainable diets in the Nordics: a scoping review for Nordic Nutrition Recommendations 2023

**DOI:** 10.29219/fnr.v68.10489

**Published:** 2024-04-15

**Authors:** Helle Margrete Meltzer, Hanna Eneroth, Maijaliisa Erkkola, Ellen Trolle, Peter Fantke, Juha Helenius, Jørgen Eivind Olesen, Merja Saarinen, Amund Maage, Trond Arild Ydersbond

**Affiliations:** 1 Division of Climate and Environmental Health, Norwegian Institute of Public Health, Oslo, Norway; 2 Swedish Food Agency, Uppsala, Sweden; 3 Department of Food and Nutrition, University of Helsinki, Helsinki, Finland; 4 Technical University of Denmark, Kgs Lyngby, Denmark; 5 Quantitative Sustainability Assessment, Department of Environmental and Resource Engineering, Technical University of Denmark, Kgs. Lyngby, Denmark; 6 Ruralia Institute, Finland & Strategic Research Programme FOOD, University of Helsinki, Helsinki, Finland; 7 Department of Agroecology, Aarhus University, Tjele, Denmark; 8 Natural Resources Institute Finland, Helsinki, Finland; 9 Institute of Marine Research, Bergen, Norway; 10 Statistics Norway, Oslo, Norway

**Keywords:** The Nordics, sustainability, diet, food production, food consumption

## Abstract

The terms ‘Nordic countries’ or ‘The Nordics’ include the five countries Denmark, Finland, Island, Norway, and Sweden. This review includes evaluation of the Nordic countries against Food and Agricultural Organisation (FAO)/World Health Organizations’ (WHO) guiding principles for healthy, sustainable diets with respect to environmental impact (principles #9 – #13) and sociocultural aspects (principles #14 – #16). A food systems perspective is taken to summarize and discuss the most important challenges and opportunities for achieving sustainable diets. Food system, food security, self-sufficiency, and resilience perspectives are applied. The information can underpin decisions when developing and implementing Food Based Dietary Guidelines (FBDG) in the Nordics.

None of the Nordic countries are on track to reach the 2030 UN climate and biodiversity goals. We describe how food production, processing, and consumption contribute to these and other environmental challenges, and what kinds of dietary changes/transitions consistent with these goals are required.

A major challenge is the high production and consumption of meat and too low consumption of fish, vegetables, and fruits. Meat production is a major source of emissions and, together with farmed fish, heavily dependent on imported feed ingredients, leaving a large land-use and water footprint in exporting countries while domestic land resources are not used optimally. Dietary patterns have changed drastically over the past 50 years, and in large parts of the population, meat consumption has doubled since the 1970s, rendering historic food culture less useful as a basis for present-day recommendations. The Nordics have Europe’s lowest use of antibiotics in animal and fish production and have made some progress in reducing food waste along the food chain. A major opportunity is better alignment of food production and consumption based on local or regional production potentials, in conjunction with better and more constructive integration with the global food system while integrating novel technologies to reduce emissions and resource use.

## Popular scientific summary

- The five Nordic countries urgently need to move towards sustainable food production and consumption.
- Associated challenges and opportunities, given the geophysical resource situation, are described in a rather comprehensive way.
- With many commonalities, but also many differences between them, it turns out all five countries need a better alignment of food consumption and production and optimizing local and regional production potentials to play a constructive role in a global, more sustainable food system.

One of the most ambitious collaboration projects between the five Nordic countries is their joint work on a regular revision and updating of the Nordic Nutrition Recommendations (1). Starting in 1980, the 2023 revision represents the sixth iteration of the process. NNR2023 provide a Nordic framework for science advice for food intake, which should form basis for national food and health authorities in the Nordic and Baltic countries in their development of national Food Based Dietary Guidelines (FBDG). Following up on the vision of the Nordic Council of Ministers to become the most sustainable and integrated region in the world by 2030 (2), the 2023 Nordic committee was mandated to integrate environmental sustainability aspects alongside nutritional and health considerations: ‘The updated Nordic Nutrition Recommendations (NNR) will therefore integrate environmental sustainability aspects into the food-based dietary guidelines, if relevant’.

All aspects of sustainability are interconnected and focusing solely on the environment is flawed, even for a complete assessment of environmental sustainability. In global food systems, environmental, social, and economic sustainability are, generally, strongly inter-dependent. Even in the relatively affluent Nordic countries, food-related environmental concerns, like greenhouse gas (GHG) emissions and biodiversity, are strongly influenced by the societal context that agriculture operates within, not least in the many countries from where the Nordics import food and feed. Sustainable local resource utilization and degree of self-sufficiency may be of vital importance to environmental sustainability, locally, regionally, and globally. However, this should not come at the cost of reduced sustainability in the exporting countries. The current paper therefore includes treatment of other sustainability perspectives most closely associated with environmental sustainability, presented in a format relevant for decision-making.

This paper is the fourth in a series of five providing background information for integrating sustainability criteria into the FBDG. The other four are the references (3–6). Further information is given in Box 1.

Box 1 Background papers for Nordic Nutrition Recommendations 2023

- This paper is one of many scoping reviews commissioned as part of the Nordic Nutrition Recommendations 2023 (NNR2023) project (1).
- The papers are included in the extended NNR2023 report but, for transparency, these scoping reviews are also published in Food & Nutrition Research.
- The scoping reviews have been peer reviewed by independent experts in the research field according to the standard procedures of the journal.
- The scoping reviews have also been subjected to public consultations (see report to be published by the NNR2023 project).
- The NNR2023 committee has served as the editorial board.
- While these papers are a main fundament, the NNR2023 committee has the sole responsibility for setting the final dietary reference values in the NNR2023 project.

A major reason for the close collaboration between the five Nordic countries, apart from being geographical neighbours, are their common values, illustrated by their ability to combine a comprehensive tax-funded welfare system with efficient public administration and a competitive business sector (7). Furthermore, the Nordic countries are among the highest ranking in international comparisons on health and welfare, as demonstrated through the Human Development Index (8), which is based on indicators such as healthy life expectancy, education and high GDP per capita. In line with this achievement, the 2023 Sustainable Development Report ranks Finland, Sweden and Denmark as the top three countries with respect to their progress toward achieving the UN Sustainable Developmental Goals (SDGs), while Norway ranks number 7 and Iceland ranks number 29 of the 166 countries reported (9). However, the report also highlights much worse performance of the Nordic countries when ‘spillover’ impacts^1^ abroad are included in the score. When those impacts are taken into consideration, Finland, Denmark, Sweden, Norway drop from their top seven positions to 128, 137, 139, and 154, respectively, and Iceland is ranked 164 (9, 10). In the report, all five Nordic countries score low on SDGs 12–15 that address ‘*… sustainable consumption and production patterns’ (SDG 12), ‘… urgent action to combat climate change and its impacts’ (SDG 13), ‘Conserve and sustainably use the oceans, seas and marine resources for sustainable development’ (SDG 14) and ‘ Protect, restore and promote sustainable use of terrestrial ecosystems’ (SDG 15)* (10). Regardless of their SDG scoring, the governments of all five Nordic countries have made ambitious strategies to meet the SDG (10).

**Fig. 1:**
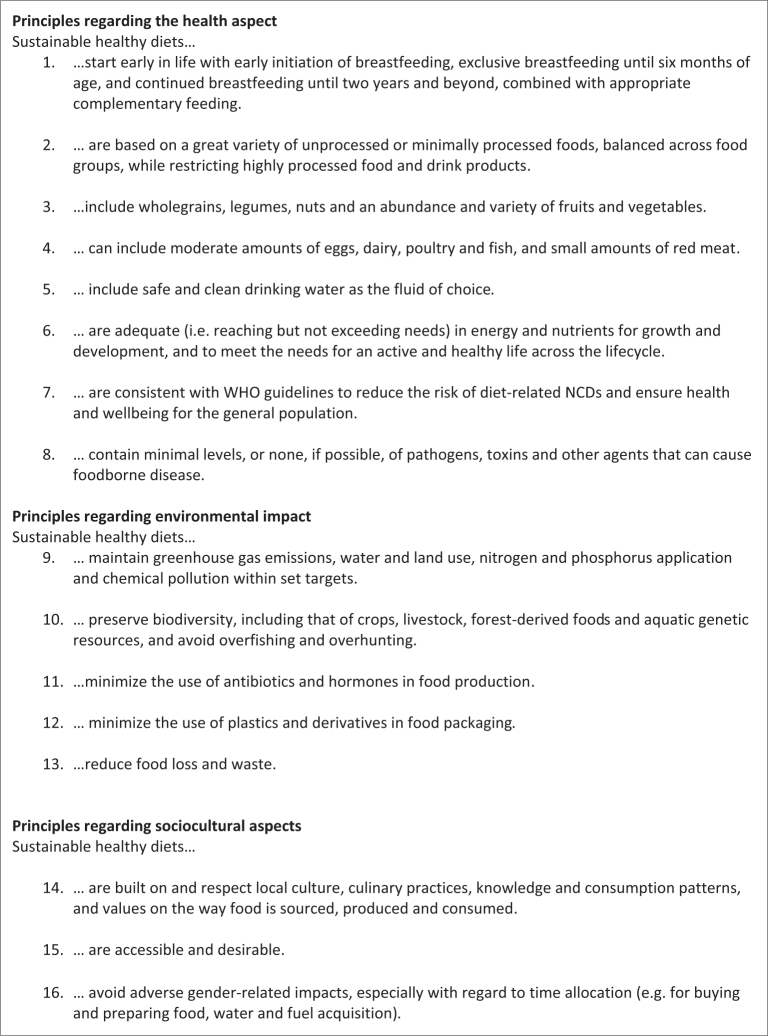
The FAO/WHO Guiding Principles for Sustainable Healthy Diets (11).

The Food and Agricultural Organisation (FAO) and the World Health Organization (WHO) have developed a list of 16 guiding principles related to sustainable healthy diets shown in Fig. 1 (11). These guiding principles define a basic set of requirements and are targeted at governments and other stakeholders in policy making and communication. They cover three categories: health, environmental, and sociocultural aspects. These guiding principles have direct implications for setting FBDG.

Globally, FBDG establish a set of conditions to help public food and nutrition, health, fisheries and agricultural policies and nutrition education programmes to foster healthy eating habits and lifestyles. They provide advice on individual foods, food groups, and dietary patterns aimed at the general public to promote overall health and prevent chronic diseases (12). Thus, they have the potential to influence both dietary habits and national food systems. To implement sustainability considerations in the Nordic FBDG, common challenges as well as country specific and local conditions must be identified, including sustainability in the countries exporting to the Nordics (13). Foremost among the common challenges is improving the dysfunctional global food system (14).

There is a need for dietary adaptations to reduce the overall environmental impact of food consumption, including, but not limited to, climate impact, land use, biodiversity loss, pollution, as well as social and economic issues. There is a similar need to develop more sustainable production and processing methods and technologies addressing the many sustainability goals (15). A given food, food group, or diet can have widely different sustainability characteristics, depending on how and where it has been produced (16, 17). Therefore, considering environmental sustainability of consumption is a necessary but not sufficient condition for a comprehensive assessment of sustainability. Here, the focus is to assess consumption in a larger food system context, including production aspects.

Worldwide, there is increased emphasis on food security, prompted by more unpredictable production and market conditions. Sustainability and agency are suggested as new dimensions, creating a six-dimensional food security framework alongside the traditional pillars of availability, access, utilization, and stability (18). According to FAO, in 2020, some 2.37 billion people – nearly one in three – faced food insecurity at the moderate or severe level (19).

Sourcing strategies is an aspect of general food system resilience, including more focus on national and regional supplies, where also productivity and global trade must be considered to diversify supply chains and increase resilience (20–22).

The aim of this paper is to discuss challenges and opportunities for adaptations to healthy and sustainable food production and consumption in the Nordic countries, given the current global food system. FAO/WHO’s guiding principles on environmental impact (#9 – #13) and sociocultural aspects (#14 – #16) of foods provide important check points (listed in Fig. 1). This paper also discusses issues of social and economic sustainability not included in the FAO/WHO principles. The health impacts (#1 – #8) of foods are scrutinized in the main NNR2023 report (1), not in this review. Box 2 gives a list of definitions and concepts used throughout the paper to help the reader through the jungle of abbreviations. Box 3 provides our key take-home messages.

Box 2 Abbreviations/concepts

- Agroecology: Agroecology is a holistic and integrated approach that simultaneously applies ecological and social concepts and principles to the design and management of sustainable agriculture and food systems. It seeks to optimize the interactions between plants, animals, humans, and the environment while also addressing the need for socially equitable food systems within which people can exercise choice over what they eat and how and where it is produced (23).
- Blue and green water: ‘Blue water’ is the liquid water in rivers, lakes, and ground water. ‘Green water’ is the water that feeds the system as rain and forms soil moisture that is absorbed by plants (and then exhaled as vapour flow) (24).
- CAP: EU’s common agricultural policy (25).
- CO_2_eq: CO_2_ Equivalents. For assessing the short-term effects of greenhouse gases, their total warming effect over a period, often 100 years, are compared to CO_2_ and summed up.
- F2F: Farm to Fork.
- FAO: Food and Agriculture Organization of the United Nations.
- FBDG: Food-based dietary guidelines.
- Food coverage: the concept pertains to the share of foods available for potential consumption in a country if no foods are exported. Thus, the difference between the degree of self-sufficiency and the degree of food coverage is whether exported food is considered in the calculations (26).
- Food security: Food security exists when all people, at all times, have physical and economic access to sufficient safe and nutritious food that meets their dietary needs and food preferences for an active and healthy life (27).
- GHG: Greenhouse gases. A GHG is a gas that absorbs and emits radiant energy within the thermal infrared range, causing the greenhouse effect. The primary greenhouse gases in Earth’s atmosphere are water vapor (H_2_O), carbon dioxide (CO_2_), methane (CH_4_), nitrous oxide (N_2_O), and ozone (O_3_). CO_2_ is by far the most important, because emissions will have large warming effects for hundreds of years. Methane is 2nd, with an initial warming effect about 100 times larger than CO_2_ but it is decomposed within about one decade. Nitrous oxide is 3rd, with a warming effect much stronger than methane. Its effect lasts a couple of centuries.
- Ha: hectare = 10,000 m^2^.
- kHa: kilo-hectares = 1,000 hectares.
- LCA: Life Cycle Assessment, an ISO-standardized environmental management tool to quantitatively assess and compare the overall environmental performance of products, services, and technologies from a broader systems perspective.
- LULUCF: Land Use, Land-Use Change and Forestry.
- Monocultures: Crops grown intensively on large fields in simplified crop rotations with low diversity. Crop rotations may enhance biodiversity, but the result on different outcomes varies between different practices such as agroforestry, intercropping, cover crops, crop rotation, or variety mixtures (28).
- Mt: megatonne = million kg.
- NCDs: Non-communicable diseases, for example, coronary heart disease, cancer, diabetes type 2, etc.
- Net zero: GHG emission regimes that do not produce further warming, that is, no increase in total radiative forcing from atmospheric greenhouse gases. For Net Zero to be sustainable, net CO_2_ emissions must be zero, and methane and nitrous oxide emissions must be lower (corresponding to emission reductions at least 0.3% per year for methane, preferably at least 1% for nitrous oxide) than the amounts eliminated from the atmosphere (29, 30).
- NNR: Nordic Nutrition Recommendations.
- NNR2023: the Nordic Nutrition Recommendations published in June 2023.
- N: Nitrogen.
- The Nordics = the five Nordic countries = Denmark, Finland, Iceland, Norway, and Sweden.
- Paludiculture: the productive land use of wet and rewetted peatlands that preserve the peat soil and thereby minimizes CO_2_ emissions and subsidence (31).
- P: Phosphorous.
- Resilience: The capacity to deal with change and continue to develop (24). In this paper, we for a large part use the concept in connection with social/ecological resilience: Social resilience is the ability of human communities to withstand and recover from stresses, such as environmental change or social, economic, or political upheaval. Resilience in societies and their life-supporting ecosystems is crucial in maintaining options for future human development (21, 24). More generally, ‘Resilience is the capacity of a social-ecological system to absorb or withstand perturbations and other stressors such that the system remains within the same regime, essentially maintaining its structure and functions’. (32).
- Scandinavia: Denmark, Norway, and Sweden.
- SDG: The UN Sustainable Developmental Goals (33).
- Self-sufficiency: Food self-sufficiency refers to a country’s capacity to meet its own food needs from domestic production. It is typically measured either by the proportion of a country’s food consumption that is met by domestic production or by per capita food production per day at the level of an adequate diet (34). It is a snapshot of the level at any given moment, not a reflection of *the ability* to provide domestic food coverage for the population, see definition of ‘food coverage’. In this paper, the degree of self-sufficiency is estimated as the percentage of calories eaten in a population that is produced domestically
- SSB: Sugar-sweetened beverages.
- UN: United Nations.
- UNEP: UN Environmental Programme.
- WHO: World Health Organization.

Box 3 Take-home messages

- The global food system is dysfunctional, currently with a large negative impact on climate, the environment, and social and health matters. Continuing business as usual may result in severe ramifications for humanity, including but not limited to, failed climate change mitigation, continued biodiversity loss, and air, water, and soil contamination. Moreover, this trajectory might exacerbate the present-day health burden and social inequalities.
- The Nordic countries, with a total population of ~ 28 mill. people, contribute to the negative environmental impacts of the global food system. All five countries score low in the global spill-over index, which indicates that they have considerable climate and environmental impacts abroad, not only domestically.
- The Nordic countries have the capacity to develop and implement sustainability-based policies for food production and consumption that may support optimal health as well as having constructive roles, both environmentally and socially, in the global food system. The countries have a large knowledge and innovation base, but public and private support for change is necessary.
- The change must and can take regional special features into account to promote global and local environmental sustainability. Decreasing the production and consumption of meat, in particular ruminant meat, is the most central issue combined with the need to increase fish, whole-grain, fruit, vegetable, and pulses consumption.
- Methane emissions from ruminants resulting from Nordic production of meat and dairy should be reduced as fast as possible. However, a holistic perspective should be applied that considers all GHGs, including methane, nitrous oxide, and changes in soil carbon. The climate impact of GHG is exacerbated by increasing emissions from peat soils drained for agricultural use.
- Both domestic and foreign land-use effects of food production need to be accounted for. This is particularly important for meat production dependent on imported feed.
- Recent Nordic use of soy in feed concentrates can cover the recommended protein supply of approximately 25 million people if consumed directly as food. As current animal protein production is resource intensive, there is a need to develop Nordic plant protein production that has an improved environmental profile contributing to global health and sustainability.
- Soy use in feeds is a prime example of food–feed competition. This food–feed competition reduces the efficiency of the existing food system, as environmental and resource costs are higher when arable land is used for animal feed production instead of directly contributing to human nutrition.
- Grazing regimes for the remaining ruminant productions need to be designed to serve biodiversity in meadows and other high nature value habitats.
- There are potentials for more local use of fish and marine products in the Nordic countries. Exploiting these and their associated resource efficiency, parts of the agricultural land presently used for feed production can be used for producing plant food to humans. This way, animal protein intake can be kept at a desired level with far less environmental impact.
- For social sustainability and justice, a wide range of healthy and high-quality food items with low environmental impacts must be made affordable, for example, fruits, vegetables, and berries, and this should become a feature of food systems at all levels. Fiscal and other food policy measures need to target this.
- Basic environmental sustainability requirements in the European context are already to an increasing extent be handled by policies and regulations like the Farm to Fork initiative. These developments need to be enforced.
- The national food systems in the Nordics have many similar traits in spite of widely differing food and agricultural policies. This indicates that there may be benefits to reap from Nordic cooperation and coordination independently of fundamental policy shifts.

## Method

Two complementary approaches were used to develop the assessment of food production and consumption in the Nordics from a sustainability perspective. Firstly, the core author team^2^ reviewed and summarised country-specific statistics, research on local aspects of the Nordic food systems, and governmental actions and initiatives. An expert elicitation was thereafter made with a larger author team, where experts provided inputs on challenges and opportunities. These are either co-authors of this paper or credited in the acknowledgement. The manuscript was thereafter subject to public consultation that resulted in many valuable inputs. Thus, the work combines knowledge gained from an overview of existing policies, scientific research literature, other relevant data, and public consultation with a comprehensive assessment of each country’s food system.

## General background, the Nordic countries

A broad outline of the Nordic countries’ food consumption and diets in relation to climate and environmental issues is given in background Paper 3 (5). We refer to this and to the Stockholm Resilience Centre report from 2019 for in-depth descriptions of food consumption in the Nordics (5, 13).

The geographical location of the five Nordic countries strongly determines the characteristics of food production in each country – mirrored in each country’s cultural food heritage. The north to south gradient for Norway, Finland, and Sweden is more than 1,840 km^3^ (55–71°N) with a substantial amount of land above the Arctic Circle, limiting the growing season in a large part of the land area. Similarly, crop production in Iceland, which lies just below the Arctic Circle, is mostly limited to the production of hay for animal feed (35, 36). At these Northern latitudes, agricultural activity is largely constrained to grasslands and dominated by dairy and meat production, including cattle, sheep, goats, and reindeer. Forests dominate large parts of the Nordic lowlands. On the other hand, the southern parts of Norway, Finland, and Sweden are relatively more suitable for growing cereals, oilseeds, legumes, sugar beets, and vegetables. Denmark is one of the world’s most intensive producers of cereals, primarily for livestock feed, and Denmark, Finland, and Sweden are net exporters of cereal grain. All the Nordic countries have large coastal waters and especially Iceland and Norway have large productive ocean areas with extensive fish production and potentials for increasing aquaculture.

Another characteristic of the Nordic countries, excluding Denmark, is that the fraction of agricultural land ranges between ~3% (Norway and Iceland) to around 7–8% for Finland and Sweden, which is far less than most mid- and southern European countries. However, crop land per capita ranges between 0.15 and 0.41 ha/person, see Table 1. Much of this land is not used very intensively today, indicating potentials for producing more food per unit land, especially as the growing season becomes longer and warmer with global warming. Constrains are unsuitable terrain and soils together with pests, drought, and flooding, which may be exacerbated by climate change. Food production in Denmark, on the other hand, resembles agricultural practices in mainland Europe with a high share of cropland (56% of land area), for further details, see Table 1. Thus, agricultural food production in the Nordic countries varies considerably and is strongly determined by population distribution, availability of suitable land, and climate and environmental conditions across the regions (Table 1).

**Table 1:** Background data on population and main characteristics of food production in the five Nordic Countries, see footnotes for references

Indicator	DK	FI	IS	NO	SE
Population (millions)^1^	5.87	5.55	0.38	5.43	10.45
Area (1,000 km^2^)^1^	42.7	303.9	103.5	323.8	447.4
Total agricultural land (ha)*	2,620	2,270	1,872	986	3,005
Cropland	2,398	2,248	121	808	2,542
Agricultural share of total area	56.2	7.4	1.2	2.5	5.7
Cropland per person (ha/capita)	0.41	0.41	0.35	0.15	0.25
Organic farmland (%)^2^	11.7	13.9	0.2	4.6	20.3
Fish production (1,000 tons)^2^					
Marine fish catches	733	141	1,020	2,451	180
Aquaculture	43	15	41	1,490	12
Marine and aquaculture for export (USD million)	4,109	194	2,010	10,797	4,345
Meat production (1,000 tons)^2^	1,886	409	34	361	567
% for export	88	15	9	<2	13
Dairy production (1,000 tons)^2^	5,666	2,407	156	1,565	2,773
% for export					13
Cereal production (1,000 tons)^2^	15,772	4,867	19	1,820	9,725
% for export	11	15	0	<1	18
Inorganic fertilizer use (kg per ha)^2^	143	93	130	208	117
Pesticide use (kg per ha)^2^	1.32	2.19	0.01	0.85	0.65
Self-sufficiency rate, % of consumed calories from domestic production	NA	80	53	40	50
Food’s cost of net family income (%)^6^	11.8	12.2	12.9	12.5	12.7
Carbon footprint of diets (CO_2_eq/year)^7^					
Meat consumption (total/red meat) (g/adult/day)^7^	161/136	145/105	117/82	147/119	110/90
Food waste per capita/year, households, including peel, skin and bones (kg)^8^	78.60	53.24	NA	77.33	60.77

All FAO data (footnote 2) are for the year 2020 as reported by the individual countries.

1 Nordic Statistics database (41);

2 FAO Statistical yearbook 2022 (42);

3 From (26);

4 From (43), corrected for imports of fodder;

5 Article on RISE web-page (44);

6 Data from Eurostat (45);

7 From (4);

8 Eurostat **Not including Svalbard.

* AGRICULTURAL LAND is land used for cultivation of crops and animal husbandry. It is the total of areas under ‘Cropland’ and ‘Permanent meadows and pastures’. CROPLAND is the land used for cultivation of crops, including grass.

The Nordic countries are self-sufficient, usually operationalized as a percentage of calories consumed that are produced domestically, to a varying degree. Denmark, for example, is a major exporter of dairy, meat, and live animals (mainly pork and pigs), while Iceland and Norway are major exporters of seafood (wild and farmed). Dairy, on the other hand, is the main agricultural export product from Finland and Sweden. Despite net export of certain foods, all five Nordic countries have high levels of food imports, with the total footprint of food production exceeding the domestic agricultural land use. This implies that substantial environmental impact on land and water use occurs outside the Nordic countries (37). For example, corrected for import of animal feed, only ~40% of calories consumed in Norway are produced within the country (38). The corresponding number for Iceland (26) and Sweden is ~50%. Finland is the most self-sufficient, with domestic food covering around 80% of calories consumed (39); data are missing for Denmark due to the large imports and exports of feed and food that make accounting of local produced food difficult. Even with a relatively high share of consumption from domestic production, such as in Finland, food production in these countries is not self-sufficient on all critical inputs, such as fertilizers and energy, and vulnerabilities exist.

In summary, the degree of self-sufficiency, reflected by net export of certain foods and import of others in the Nordic countries, is highly dependent on the import of food for human consumption, feed for animal production, and other input imports for agriculture and aquaculture. This highlights the importance of accounting for the environmental impact abroad when evaluating sustainability and food security in the Nordic countries. As this accounting will be done differently according to the perspective applied, ‘self-sufficiency’, as a single number, is not well defined (40).

## The Nordics evaluated through the lens of FAO/WHOs guiding principles

### Environmental aspects

Here we evaluate the food system in the Nordics against FAO/WHO’s guiding principles # 9–16 (Fig. 1). The following text gives an overview of the challenges and opportunities. Table 3 gives examples of the individual Nordic countries’ specific challenges and opportunities.

### Principle # 9: Maintain greenhouse gas (GHG) emissions, water and land use, nitrogen and phosphorus application, and chemical pollution within set targets

#### Obligations at the EU level: Farm to Fork and climate neutrality

The European Union has through its Farm to Fork Strategy (F2F) set a number of overarching environmental goals for all EU members, including Denmark, Finland, and Sweden (46). While a discussion of concepts, definitions, etc. in this context might be highly relevant, it is outside the scope of the present paper. It should be noted that though the original F2F proposal may be watered down in the political process, the main goals are likely to persist, but in a longer time frame. The following are relevant for #9.

- Developing a carbon farming scheme to support soil carbon sequestration.
- Cutting the use of chemical pesticides by half by 2030.
- Reducing soil nutrient losses by 50% by 2030 while ensuring no loss of soil fertility.
- Reducing the usage of chemical fertilisers by 20% by 2030.
- Transitioning 25% of all member state agricultural land to organic by 2030.

In addition, EU’s goal of carbon neutrality by 2050 impacts all the Nordic countries. The EU aims to reduce the GHG emissions 55% by 2030 (compared to the 1990 level) and be climate-neutral by 2050, having an economy with net-zero GHG emissions (47). In the Nordic countries, stricter carbon neutrality targets have also been set in terms of time. The IPCC summarizes the essential conditions: ‘reaching and sustaining net-zero global anthropogenic CO_2_ emissions and declining net non-CO_2_ radiative forcing would halt anthropogenic global warming on multi-decadal timescales’ (48).

These obligations should, if measures are implemented properly and aligned with interest groups and political parties, over time go a long way towards fulfilling #9’s GHG ambition. It must, however, be noted that the implementation has been problematized by farmers. These goals and timelines for research on carbon neutrality in agriculture and food production are extremely ambitious and require massive investments in research, innovation, and implementation of sustainable production and processing systems along the many food value chains (15, 49, 50).

#### Common Nordic challenges and opportunities connected to Principle # 9

The most crucial environmental challenges related to food production mainly take place at the production stage within agriculture, fisheries, and aquaculture rather than in the subsequent processing industry or elsewhere in the value chain (51). These challenges include high GHG emissions, the decline in species diversity in farming environments, leakages of nitrogen and phosphorus, together with sediment and organic matter loading, in agriculture through ditches and subsurface drainage, and environmental pollution from a wide range of agricultural pesticides (37, 52).

*Dietary patterns and global food production.* Unhealthy diets contribute to obesity and diet-related chronic diseases that come at a high cost to the individual and public sector. These health aspects are of concern in the Nordics too. In addition, the current Nordic food consumption pattern, characterized by high consumption of foods of animal origin and limited self-sufficiency for certain foods such as fruits and vegetables, implies extensive food and feed import. This makes it challenging to fully meet the conditions set out in principle #9. For example, imports without efficient certification systems may, directly or indirectly, lead to biodiversity-rich natural ecosystems being converted to cropland or pastures in exporting countries. It should be noted that more local production and less imports would provide more control over sustainability aspects, and in some respects, like antibiotics use, animal welfare, and pesticide use, Nordic practices score much better than average. However, this does not necessarily imply that, generally, local Nordic production is more sustainable. The land use issue is most pronounced with meat, farmed fish, and dairy, but the import of high-quality protein for animal feed increases the pressure on land use and leads to unnecessarily high emissions both in exporting and importing countries (53–55). For example, the fraction of the current import of soy for animal feed for domestic consumption in Denmark (Danish meat/dairy consumption) alone would cover more than 100% of the population’s basic protein needs (56).^4^ In Norway, the corresponding figure is over 50%, while a much smaller amount of high-quality protein would be needed for efficient use of domestic grass and grain feed resources (57).

Compared to agriculture, many fisheries and aquaculture systems can produce proteins with lower GHG emissions than beef (but similar to those of chicken and eggs) and with lower impact on other environmental stressors, so optimal resource utilization is important. Over-fishing and specific impacts on aquatic ecosystems may make the impact higher (16, 58). Within capture fisheries, currently, the use of fuel relative to the amount caught is the key driver. Small pelagic species like herring have in relative terms the lowest GHG emission, while fishery for flounders, halibuts, farmed shrimps, and soles have the highest GHG emission (59). Other species like cod, hake and haddocks, and farmed salmon are in between. In a report from European scientists delivered in 2017, they claim that the only way to obtain significantly more food and biomass sustainably from the ocean is to harvest seafood that on average is from a lower trophic level than we currently harvest. Thus, they have openings for zooplankton, krill, and mesopelagics as important new resources from marine harvesting (60). For aquaculture, the production of feed ingredients is a major source of GHG emissions as well as being a source of other stressors (see principle # 10).

*Greenhouse gas emissions.* There are several inherent goal conflicts involving GHG that the Nordic countries are faced with: Foremost, population growth and generally high demand for meat may make large percent-wise reductions in GHG emissions challenging, at least in the short term, if the present-day emphasis on ruminant meat is continued. In the longer term, ongoing work on reducing enteric methane production in ruminants as well as other farm sources of methane and nitrous oxide emissions combined with enhanced soil carbon storage may alleviate this situation by reducing net emissions by more than 50% by 2030 (15).

All five Nordic countries have challenges with CO_2_ and nitrous oxide emissions from cultivated peatlands (organic soils), which might require farm-specific solutions and novel types of collaboration with farmers to fully resolve (61–63) (Table 2). Such areas account for about 25 Mt of CO_2_ emissions, which are accounted under land use and land use change and therefore not fully included in many emission reports. Mitigation projects are ongoing in all five Nordic the countries, including taking areas out of production, restoring wetlands, and moratorium on peatland cultivation, or change to paludiculture or other forms of production with reduced emissions. The high CO_2_ emissions from organic soils represent a larger and more acute problem than the nitrous oxide and methane emissions combined in some Nordic countries (64).

**Table 2:** Agriculturally sourced (including land use) greenhouse gas emissions and (FAOSTAT 2023) (71) efficiency of nitrogen and phosphorous application

Indicator	DK	FI	IS	NO	SE	Total
Agricultural GHG emissions, Mtons CO_2_-equivalents						
Methane^1^	6.01	2.11	0.30	2.54	3.19	14.15
Nitrous oxide^1^	4.39	3.77	0.34	2.66	4.11	15.27
CO_2_ from cultivated peatland^2–7^	4.92	7.53	1.80	1.80	8.69	24.74
Cultivated peatland, 1,000 ha^3–7^	170	260	62	62	300	854
Agric area 1,000 ha	2,620	2,270	1,872	986	3,005	10,753
Methane, tons CO_2_-eq/ha	2.29	0.93	0.16	2.58	1.06	1.32
Nitrous oxide, tons CO_2_-eq/ha	1.68	1.66	0.18	2.7	1.37	1.42
N, Synthetic fertilizer kg/ha	95.6	45.4	72.8	132.2	82.2	
N, Manure kg/ha	71.7	13.3	33.4	58.9	24.9	
N, Crop removal kg/ha	91.7	33.0	NA	32.5	55.9	
N, Crop/Fertilizer %	54.8	56.3	NA	17.0	52.2	
P, Synthetic fertilizer kg/ha	6.0	5.2	16.7	11.4	6.9	
P, Manure kg/ha	22.5	4.9	7.5	14.4	6.4	
P, Crop removal kg/ha	17.9	6.1	NA	6.2	10.9	
P, Crop/Fertilizer %	62.8	60.8	NA	23.8	81.5	

1 World Resources Institute (+ ref-number),

2 IPCC 2014 (+ref number)

3 From (+ref number Kløve et al 2017)

4 From (ref Regina et al 2019)

5 From (ref number Soil conservation Service of Iceland)

6 From (+ ref number for Farstad et al 2020)

7 From (+ ref number for Olsson et al 2015)

*Progress towards national targets for agricultural GHG emission reduction.* According to the Danish Climate Council, Denmark is not on track to reach its goals of 70% total reduction in 2030 compared with 1990 and net zero in 2045, in part because of lagging reductions in agricultural emissions. In 2019, agricultural emissions accounted for about one third of the total Danish emissions (15). In 2021, The Danish parliament decided on a detailed plan to achieve the target for 2030, including reducing methane and nitrous oxide emissions from agriculture, rewetting organic soils, and enhancing soil carbon through use of biochar (65). However, additional initiatives are needed, such as specific climate taxes in agriculture and subsequent changes in the type of agricultural production (66). Finland has a target of 29% reductions in agricultural emissions, but only about 6% are planned in CAP (67). There is a heavy political emphasis on reducing emissions from use of peat soils for farming in Finland. These are accounted for in the LULUCF and make up more emissions than all the agricultural emissions together. The goal for emissions reduction from agriculture according to Iceland’s action plan is 5% in 2030 relative to the emissions in 2005 (68). Norway aims at a large reduction in agricultural emissions in the period 2021–2030, which may be enhanced by dietary changes (69). Those changes have not yet started to materialize (38, 39). However, Norwegian agricultural organizations have proposed an eight-point plan for reduction that does not involve dietary changes (70). Sweden aims for net zero in 2045, and GHG emissions from agriculture are estimated to be reduced to 6.2 Mt of CO_2_eq in 2030 because of more efficient cattle production. This may, however, be counteracted by national goals of increased agricultural output.

Shifting to sustainable (i.e. considering what’s included under Principle # 9), balanced diets and reducing over-consumption was listed in the 6th IPCC assessment report as an important measure to reduce climate impact (72). The Emissions Gap Report also mentions demand-side dietary changes as a key issue (73). One reason for the discrepancy between goals and policy measures may be that the necessary dietary changes to achieve the climate goals are considered potentially problematic for agriculture. There is a concern that significantly reduced domestic meat and dairy consumption might lead to reduced utilization of local resources, both grass and grain based. There is a globally increasing trend toward increased meat consumption in developing countries, and any reductions in domestic meat consumption may therefore be offset by increased exports, in particular for productions that already have this focus, such as Danish agriculture, which for a long time has been export-oriented, and thus less sensitive to changes in domestic consumption patterns. This demonstrates that the potential problems may, in principle, be solved by better integration with the global food system, which will also reduce the global GHG emissions resulting from a given total consumption (74).

Be it locally sourced or from the global market, meat and to a somewhat lesser extent dairy, contribute to sustainability challenges. In addition to the need for reducing methane emissions from enteric fermentation and manure, much grain and legumes that could also be used for food are used for fodder, thus food-feed competition is involved. In addition, the use of feeds may be far from optimizing the total food supply, feed-feed competition may also be relevant. This may or may not represent good practices, depending on several factors, like fodder influence on methane production and the global food supply. The global food supply situation is widely expected to become more stressed in the future (75, 76)

*Future aspects:* The current rate of warming in the Nordics is 0.25–0.5 degrees/decade, generally somewhat less in the growing season (77). This opens new opportunities for agricultural diversification and increased yields. Increased use of winter cereals in the Nordics will contribute to boosting yields. However, climate changes are, generally, also associated with more extreme and variable weather that risks higher variability in crop production due to for example drought or flooding. In addition, there is a risk of higher prevalence of plant diseases, new types of pests, and competition with invasive species (78). The climate change will also affect the species distribution of fisheries resources.

*Water and land use.* Agriculture in the Nordics is mostly rainfed. However, some parts of the more intensive agriculture in the Nordics are irrigated from groundwater and water reservoirs that are replenished every year. This, therefore, does not constitute a threat to water availability. Progressing climate change may challenge this (79), for example, as timing of rains may increasingly diverge from what is needed in the rainfed farming. Some of the imported food and feed may be based on cropping systems with overuse of water for irrigation. As mentioned above, the agricultural land use in the Nordics varies greatly, with widely differing effects on environment, climate, and biodiversity.

*Fertilizer use and nutrient losses.* There is both globally and in the Nordics a loading of nutrients (nitrogen and phosphorus) from agricultural systems to the atmosphere, groundwater, and aquatic ecosystems. In the Nordics, this negatively affects the coastal waters and Baltic Sea (80). There are regulatory frameworks in place, such as the EU Water Framework Directive and the National Emissions Ceiling Directive, but they have so far shown to be ineffective in reaching environmental targets (81).

*Pesticide use and its contribution to chemical pollution.* A wide range of chemical pesticides is used on crops produced in the Nordics, complemented with pesticides used elsewhere on crops that are imported into the Nordic countries. This pesticide use is a main contributor to global chemical pollution that is projected to exceed the related Planetary Boundary (82, 83). On crops grown in the Nordics, an estimated 10,000 tonnes of chemical pesticides were applied in 2020 (https://fao.org/faostat), with the highest doses applied to fruits and vegetables (84). This may constitute a challenge for moving from meat to vegetable and fruit consumption, as pesticide use may potentially increase. For cereals and pulses, the challenge should be smaller, as much of the change would be from feed to food use, and human pesticide exposure from such sources is generally low. This should foster innovation in crop production with reduced pesticide use, based on, for example, chemical substitution (85) and other, more sustainable pest control solutions, involving integrated pest management (IPM), and even organic farming, where chemical pesticides are either prohibited or used in smaller doses (86).

*Global food system aspects related to principle #9.* As mentioned above, many potential problems associated with necessary changes in Nordic patterns of production and consumption may, in principle, be solved by better integration with the global food system. One important factor is the type of animal feed used. There is an ongoing process with more systematic use of industrial biomass side-streams, like press cakes from rapeseed oil production, cuts and molasses from sugar beet processing and by-products of bioethanol production, and possibly, better food waste utilization. In Denmark, there are also ongoing efforts to upscale biorefining of grass for protein for livestock feed as a substitute for imported soymeal, and this may be achieved with reduced overall land use (87). This may ease the pressure on feed components that have many alternative uses, foremost as human food (88). The political goals of the circular economy are compatible with the climate goals and highly relevant to the food and nutrition sector. At the farm level, relatively modest amounts of phosphorous, potassium, trace elements, etc. are usually sufficient to compensate for the loss in crops. Thus, re-configuring the food system for a higher degree of circularity, on all scales, is highly desirable (89).

### Principle #10 Preserve biodiversity, including that of crops, livestock, forest-derived foods, and aquatic genetic resources and avoid overfishing and overhunting

The five Nordic countries are committed to the fulfilment of the Aichi targets on biodiversity and their recent follow-up (90), which will require greater investment in the extent and management of protected areas on land and sea, as well as enhanced biodiversity conservation and management across the economy (91). The Svalbard Global Seed Vault, housed on the Norwegian island of Spitsbergen, is a globally significant effort to protect and secure the world’s biological and seed diversity for food and feed crops in perpetuity (92). This type of ex-situ conservation of germplasms is important, but equally, in-situ conservation of food plant varieties and farm animal breeds by sustaining farming of even old varieties and breeds, and use of these as food, significantly contributes to goals of preserving genetic resources (93–95).

After a century of specialisation and intensification of agriculture, the local biodiversity associated with traditional agricultural landscapes is threatened in many places in all five Nordic countries – much of the same applies to agriculture that supports Nordic imports. This is driven by a range of factors, including large-scale mechanisation, leading to landscapes with less boundaries and places of refuge for insects and birds. Increased use of agricultural inputs leads to extended areas of crop monocultures, leaving little room for wildlife in the farmland. For some of the countries, there has been a reduction in grazing livestock due to decreasing profitability. These factors have to some extent been counteracted by increased focus on variants of organic farming that emphasizes integration of livestock and less use of agricultural inputs. Countermeasures that also enhance climate change adaptation can include increased focus on crop wild relative conservation planning (96, 97). There is a trade-off between a biodiversity focus and mitigating increased GHG emissions. Optimally, sustainable solutions should not undermine each other but contribute to each other.

The degree of threats to biodiversity varies across countries and with country-specific aspects. In Iceland, large-scale livestock grazing and historical woodland clearing under cold climatic conditions and frequent volcanic activity have resulted in dramatic ecosystem degradation throughout much of the country (35, 98). In Finland, Norway, and Sweden, the situation is almost the opposite, where abandonment of grazing in semi-natural pastures is threatening many red-listed species, with risk of extinction because grazing animals have been removed from the landscapes (99–102). In Denmark, there is also too little grazing of extensive and semi-natural pastures resulting in declining biodiversity due to non-profitable schemes to support grazing (103). These pastures are examples of agricultural systems where human interference is crucial for maintaining a high level of biodiversity – in this case, keeping grazing animals on high-nature value grasslands (104). If these lands are abandoned or planted with forest, numerous species will be threatened. Thus, grazing ruminants linked to these grasslands can support biodiversity, and in, for example, Sweden there are relatively many of these landscapes left (99, 105).

Generally, rotating cattle, sheep, goat, or other grazing livestock between different pastures can improve both soil health and plant and insect biodiversity (106–108). It must, however, be noted that too high grazing pressure will degrade the soil and ecosystem, as has happened in Iceland, and that different grazing practices can result in ecosystem services of different quality. Arguably, ecosystem services have had rather low priority in the shaping of Nordic agriculture, and the current livestock size is enough for a much wider use of pastures than the current agricultural structure can provide. For example, the average number of milking cows on Swedish dairy farms is around 100. Thus, there are too few herds, making it impossible to match herds well with a large number of pasture areas. With a structure similar to Austria (average 20 cows), pastures could be much more widely used. Reducing the amount of feed concentrate in the cows’ rations, 30–50% of energy needed in the Nordics today, to, for example, 20–25%, typical for organic milk production, would also allow for much more grazing with the current livestock size (109). Such large-scale restructuring of milk production would of course be difficult and slow to carry out in practice and would probably need considerable economic support, and it may also lower production efficiency, challenging some of the other sustainability objectives such as land use, emission reduction, and nutrient losses.

The most important constraints on the exploitation of Nordic grass resources are winter feed supply and caps on GHG emissions. Together, they limit the ruminant population to a small fraction of what summer feed supply allows for. This situation seems to apply in most of the Nordics, and therefore preserving biodiversity by grazing livestock should be viewed mostly as an opportunity, with the farm structure and associated costs representing the biggest challenge.

Organic farming serves in maintaining higher species diversity in agricultural landscapes than conventional farming. This results from several practices that characterize organic farming, such as lower rates of nutrient applications, use of organic fertilizers, diverse crop rotations, and crop-livestock integration (110, 111). However, the lower per-unit-area productivity of many organic farming systems compared to conventional may increase the reliance of food and feed imports from elsewhere (112), or require larger land areas for production, with potential negative biodiversity impacts. More research on these aspects is warranted.

Foods and animal feed consumed in the Nordics have a biodiversity impact in the countries where they are produced. This impact varies according to region, production methods, and land use history. Biodiversity impacts are highly dynamic and site specific. Large impacts come from imported foods and feeds. Particularly products that are known drivers of deforestation in tropical regions, such as palm oil, coffee and cacao, and feedstuffs, like soy, may have a large negative impact on biodiversity abroad (105, 113). The total effects will vary with production regimes, import volume, etc. For example, soy is considered the most problematic ingredient in Norwegian animal feed although the country only imports certified, non-GMO soy from Brazil for domestic animal feed, about 7.5% of total feed concentrate consumption (114).

Fisheries resources in the North Atlantic are mainly well regulated and sustainably harvested based on the management advice from the International Council for Exploration of the Seas (www.ices.dk). For example, the North Atlantic is the major area for high volume of cod harvested and going to the international markets, including as the important bacalao ingredient. However, several smaller fisheries, locally very important, have had serious problems, including the whole stock, in both eastern and western Baltic Sea and also parts of the North Sea. The Baltic cod stock lost its MSC certification in 2015 because of declining stocks and the decline has continued into 2020 when the Total Allowable Catch (TAC) was set at zero. For consumers, this shows the importance of origin tracing of commercial fish species that can be sustainably harvested in some areas but might be overfished in others. The reason for the collapse in the Baltic cod is probably a mixture of overfishing and a row of other environmental factors such as pollution and climate change (115). It is very important that the fisheries quota and actual fisheries are reduced fast enough if stocks are falling, whatever the reason for the reduction is.

When it comes to aquaculture, negative impacts on biodiversity are to a large extent caused by land transformation and unsustainable fishing for feed ingredients (54). Freshwater use can be high in the smolt production, and increasing use of RAS (Recirculating Aquaculture) is introduced to reduce freshwater use. The environmental impacts are also mainly related to feeds (55, 58), but release of surplus nutrients and other waste products may be of most concern at farm sites. The large volume of farmed salmon and trout compared to wild stocks can be a challenge to biodiversity through escapes and increase in the number of salmon lice on the wild fish populations. Aquaculture of non-fed species – like mussels and seaweeds – has the smallest environmental impact of all seafood and can also provide environmental services (i.e. nutrient absorption and removal) (116). Capture fisheries can also result in negative impacts on aquatic food webs and biodiversity, where both how much is being fished and the type of gears involved are of importance (58). Some fishing methods such as bottom trawling can give harmful effects on marine ecosystems including soft bottom and deep-water corals. On the other hand, capture fishing, especially for small fish such as Baltic herring, can also remove nutrients from the water and thus help in the fight against eutrophication (117) and its consequences for biodiversity. This method is used in water management, but from a food perspective, the challenge remains the use of fish catch for human consumption. It may require the development of new fish products.

### Principle #11 Minimize the use of antibiotics and hormones in food production

One of many goals of the Farm to Fork Strategy of the European Union is related to principle #11: ‘Reducing total EU sales of antibiotics for farmed animals and aquaculture by 50 percent’ (46).

Between 1999 and 2006, the EU phased out the use of all antibiotic growth promoters, and since the 1st January 2006 no antibiotics have been licensed for growth promotion. However, the ban that was introduced in 2006 did not apply to imports from third countries; this came into place with the 2019/6 regulation (118).

In January 2022, the European Union banned all forms of routine farm antibiotic use, including prophylactic group treatments. Using antibiotics to compensate for inadequate husbandry or poor hygiene also became illegal. If properly implemented, it should lead to a large reduction in farm antibiotic use, help tackle the serious crisis of increasing antibiotic resistance, and protect human and animal health (118).

It is noteworthy that some aquaculture production of fish, for example, salmon production in Norway, is done with minimal use of antibiotics because of the development of vaccines during the 90-ties. Many farmed aquatic species still lack efficient vaccines, and antibiotics belonging to the group of critical antibiotics for human healthcare are used throughout the world (119).

**Fig. 2:**
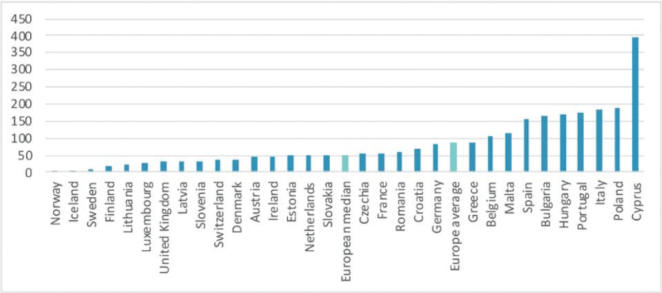
Veterinary antibiotic use in Europe in 2020 (mg per kg of PCU)*. From (125) based on data from (126). *PCU = mg of active substance per population correction unit, where PKU corresponds approximately to the total weight of live animals in a country, expressed in kg.

The five Nordic countries differ significantly from the rest of Europe and other continents when it comes to the use of antibiotics in livestock production, see Figure 2. In general, little antibiotics are used, particularly in Norway, Iceland, and Sweden (Figure 2). On the other hand, the estimates, when used for exposure assessment, might be skewed by the fact that meat is imported from productions with intensive antibiotic use – but data are not available to correct for such imports. The development of antibiotic resistance is a threatening slow-growing pandemic that is expected to have dramatic consequences if the global development is not reversed quickly.

The same strict laws on antibiotic use apply to farmed fish, where the use of antibiotics is extremely low compared to the protein production (120). It should be noted that during 2014 to 2016, Norway and Chile accounted for 53 ± 3% and 35 ± 3% of global production, respectively, and administered 0.06% ± 0.02% and 96 ± 0.09% of antimicrobials used in global salmon farming (121).

The low overall incidence of antibiotic resistance in the Nordic countries and the relatively low consumption of antibiotics in food production can be attributed to a joint effort by the primary industries, authorities, and research to prevent disease rather than treating diseases in animals and fish. In a more sustainable food production, this approach must be central as part of the general principle of strengthening links between soil health, plant health, fish health, animal health, and public health.

### Principle #12 Minimize the use of plastics and derivatives in food packaging

FBDG do not usually address environmental issues related to food packaging. Food contact materials may contain compounds that are suspected to adversely affect health (122, 123), and unnecessary plastic pollutes the environment if not handled adequately (124). However, there is still a role for plastic in food packaging, as plastic wrappings increase the lifespan of many fresh food products and thus reduces food waste (127, 128). Use of alternative wrapping materials is expanding and gradually taken into use. The European Commission has a number of initiatives to ensure reusable packaging options, get rid of unnecessary packaging, limit overpackaging, and provide clear labels to support correct recycling, all to ensure that the packaging sector will be on track for climate neutrality by 2050 (129).

A number of disposable plastic items have since 2021 been prohibited according to EU regulation (130). The legislation prohibits certain single-use plastic items on the market, thereby implementing the EU directive No 2019/904 on the reduction of the impact of certain plastic products on the environment. These items include cutlery, plates, straws, beverage stirrers, and food and beverage container and cups made of expanded polystyrene. This EU directive has also been implemented in Iceland and Norway. Research shows that only a holistic approach can guide us toward the most sustainable waste management systems, but more research is needed to support this (131).

The Nordic Ministers of Environment and Climate have actively worked for a legally binding global agreement on plastic pollution (132).

### Principle #13 Reduce food loss and waste

Dietary changes, technical changes in food production and processing, legislative changes, and reduction of losses and waste are all necessary for the food system to fit within planetary boundaries (133, 134). Approximately 3.5 million tons of food are wasted each year across the Nordic region. All countries have committed to halving waste by 2030, whether through government-led initiatives, public or private partnerships or voluntary, multi-stakeholder initiatives such as Denmark’s national awareness-raising campaign, ‘Stop Spild Af Mad’ or Norway’s ‘Bransjeavtalen om reduksjon av matsvinn’ (135). Also in Sweden, authorities work together to stop food waste (136).

It varies between the Nordic countries where food losses and waste are largest, that is, in households, or processing and manufacturing or restaurants and food services (Table 3). For example, in Finland, Silvennoinen et al. found that the average amount of food waste is between 53.0 to 62.1 kg/cap/year, in which the amount of originally edible food is between 23.0 to 28.4 kg/cap/year (137). It is rather low compared to other Nordic countries (Table 1). Although being an issue of wasted materials and in a way, environmental costs for no gain, according to the Finnish dietary assessment, consumer’s food waste accounted for only 4% of dietary climate impact (138). Thus, food waste was identified in Finland to be less important measure for consumers to reduce the climate impact of diet than the dietary changes and choosing the best products within product categories that could give incentive to food chains to improve their performance (138, 139). Similar findings apply to Norway, where postproduction food waste emissions have been estimated to account to 10% of overall food consumption emissions (140). Waste levels vary with food categories, with higher levels of waste in lower emission categories (bread, milk, vegetables), and vice versa; meat is less wasted.

**Table 3:** Examples of challenges and opportunities for the five Nordic countries according to Principles #9 – 16 in the FAO/WHO Guiding Principles for Sustainable Healthy Diets (11) described in Fig. 1

Denmark	Finland	Iceland	Norway	Sweden
**Challenges**				
Principle # 9: Maintain **a)** greenhouse gas (GHG) emissions, **b)** water and **c)** land use, **d)** nitrogen, and **e)** phosphorus application, and **f)** chemical pollution within set targets (interpreted as targets within a country).				
Cross country: None of the countries on track to reach the 2030 SDG environmental goals. All countries have a large spill-over effect because of food and feed imports. Drained peatlands due to agricultural land use change are major emitters of GHG.				
a) Implementation of the government plan to substantially reduce agricultural GHG emissions and rewet drained peatlands is going slower than anticipated and is needed to meet goals. Changes in consumption (including reducing consumption of meat and dairy) are also needed (66). b) Polluted groundwater levels (pesticides, pharmaceuticals, nutrients, metals) (229) c) High dependency on imported vegetables and legumes. Most land is used for feed crops to support animal-based food exports. d) – e) Agricultural activities have contributed to nutrient pollution of coastal waters (230) and of groundwater with nitrates (231). f) Pesticide residues are found in food, mainly fruits and vegetables (84). In general complying with applicable regulations, but a few excesses (232). Domestically and organic grown products have low or no content.	a) Not on track (233). Drained peatlands cause major emissions (234, 235). Animal-source proteins, primarily from meat and milk, dominate the protein supply (138). b) Not a challenge c) Cultivated peatlands are a major challenge (235). A downward trend in agricultural land use resulting from reduced livestock production. d-f) Only partly tackled. Major pollution of agricultural N and P to Baltic Sea (235)	a) Not on track, LULUCF accounts for 2/3 of total emissions (68). Large subsidises to meat but not to plant production (35, 98). b) Not a major challenge c) Soil erosion and over-grazing is a major challenge, but also drained wetlands (35, 236). d) Not a challenge e) Not a challenge f) Not a challenge	a) Not on track, too high consumption of meat and dairy. Approx. 90% of arable land are used for meat production directly or indirectly (49). b) Historically not a challenge, but severe drought in Eastern Norway in 2018 and spring 2023 more than halved harvests. c) Too much soil suitable for human food production is converted to grassland for fodder (237). Arable land also threatened by other areal changes. d) – e) High eutrophication in inner coastal areas due to overuse of N and P (238). f) Pesticide residues both in domestic and imported foods remains a challenge (239).	a) Not on track to reach the national objectives on climate (240) b) Availability and water quality is an issue in certain regions and identified as a future risk associated with climate change. c) Overall, a reduction in land used for agriculture. Drained peatlands accounts for 20% of territorial GHG emissions (241) d) – e) Generally, emissions of agricultural N and P to the Baltic Sea and lakes are below set maximum levels but remain a challenge (242).
Principle # 10: Preserve biodiversity, including that of crops, livestock, forest-derived foods and aquatic genetic resources, and avoid overfishing and overhunting.				
Cross country: Imported foods impact biodiversity loss in the countries of origin, for example, coffee, cocoa, and bananas. None of the countries seem to be on track to reach the EU Biodiversity strategy goal of 30% protection (hereof 10% highly protected) within 2030 (243, 244).				
Declining biodiversity in the agricultural landscape, mostly due to increase in intensive agricultural production, chemical pollution, and too little grazing (245). Maximum 2.3% of the land area is protected, while additional 5.3% may be assessed as protected after a more detailed process of evaluation.	Species decline in rural biotopes due to changes in agricultural practises. Large impact on biodiversity declines abroad through imported foods, for example, soy for fodder (246, 247).	Dramatic ecosystem degradation due to livestock over-grazing (35). - Increase of traditionally grown and wild vegetation for food is a challenge but boosts preserving of biodiversity. - Land-use of imported foods often impacts biodiversity loss in the countries of origin.	Land-use of imported foods and feeds impacts biodiversity loss in the countries of origin. - Intensive agriculture, monocultures, and domestic land use change are the largest inland threats (248, 249). - Within aquaculture, land use of imported feed (e.g. soy) impacts land use and biodiversity in the countries of origin (54). - Waste from fish farming (faeces, feed spillovers, etc.) influences the seabed (250). - Bottom trawling impacts the sea floor (212).	The agricultural landscape’s ecosystem services are in generally good state but not secured on a long-term basis and the Swedish environmental objectives related to biodiversity cannot be reached with current means of control (102). - Too high consumption of certain seafoods may contribute to biodiversity loss (251).
Principle #11: Minimize the use of **a)** antibiotics and **b**) hormones in food production.				
Cross country: All five Nordic countries are in the forefront in low use of antibiotics, Norway, Iceland, and Sweden ranking lowest in Europe (121). The achievement is the result of good collaboration between primary industries, authorities, and research. Hormones are forbidden by EU law; the prohibition applies to member states and imports from third countries alike (252)				
Principle #12: Minimize the use of plastics and derivatives in food packaging				
Cross country: Alike most other countries, also the Nordics have plastics and their chemical components integrated in all areas of daily lives, with plastic pollution being a major challenge, not least in marine environments (253). A recently published Nordic report on the issue illustrates the Nordic countries will to support common global rules set out in an international, legally binding instrument on ending plastic pollution (254).				
Principle #13: Reduce food loss and waste				
Cross country: Food loss and waste is a major challenge in all Nordic countries, with household waste being the largest contributor, see Table 1 for gross household waste per country, which includes peel, skin, and bones.				
The food loss in Denmark is about 540,000 tons per year (255). About 46% of the loss is in the household and the rest in the primary production, food industry, and retail.	Total amount of food waste in the Finnish food chain is about 641 million kg/year. The amount of originally edible food waste is estimated to be about 361 million kg/year (256).	Iceland has an action plan on behalf of the government to diminish food waste. A large study and plan for further action is on-going (257).	Total food loss and waste is approx. 450,000 tons in 2021. Almost half came from households (equivalent to ~ 40kg/person/year), 19% from the food industry, 14% from food retail sector and 9% from agriculture (258). Agreement on 50% reduction by 2030 (135).	Household food loss and waste was about 619,000 tonnes in 2021 ~ 59 kg/person/year, about 26% of that is estimated to be avoidable food waste (259).
Principle #14: Are built on and respect **a**) local culture, culinary practices, knowledge, and consumption patterns, and **b)** values on the way food is sourced, produced, and consumed.				
Cross country: Culinary practices are a moving target and very diverse, thus what is local culture is challenging to capture. All five countries have a common history of high milk and dairy consumption and are presently among the world top 15 regarding milk consumption/capita (260). Existing dietary habits and individuals’ resistance to change slow down transformation to sustainable food system (261).				
The prevalence of vegetarian diets has increased recently, although still on a very low level. The average diet is still high in meat and relatively high in milk and cheese. Plant-based meat and milk alternatives increased by 46% from 2018 to 2019 but stagnated in 2020 (262).	The prevalence of plant-dominated diets remains modest (182). Food culture, traditions and identity are deeply rooted in milk and meat production and consumption, presently having the 2nd largest consumption of milk in the world (98 kg/person/year).	Knowledge on current dietary habits is based on a new study making comparison to a couple of former studies available (263). Plant-based diet is more favoured among younger than older people. New ways are both welcome and not, depending on the group, but the average intake of, for example, red meat is still high (263)	Food culture has changed dramatically over the last 100 years (264, 265), illustrated by Norway having the weird record of having the largest consumption of SSB and frozen pizza per capita in the world (ref) Too few eat according to FBDG, for example, only ~ 15% eat 3 potions vegetables and 2 fruits/day (266).	Consumption patterns are lower than current recommendations on vegetables, fruits and whole grain while higher in sweets and snacks and red and processed meat (267).
#15: Are accessible and desirable.				
Cross country: Food costs as share of total expenses of households is an issue in all five countries. Socioeconomic status influences the ability to access and purchase healthy foods. Overconsumption of convenience foods high in energy and low in nutrients is a challenge.				
Social inequality with regard to unhealthy dietary patterns is seen in both men and women in 2021. The relative inequality decreased from 2010 for both genders, but there was an increase in absolute inequality (268). Few people eat according to the FBDG (DANSDA 2011–2013). However, it may not be more expensive to eat according to the FBDG than the current omnivore average diet (172).	Food consumption, the sustainability of food choices, and food security differ by sociodemographic groups, including income level, education level, gender, and place of residence (157, 269–271). The food retail market is dominated by two domestic chains (the ‘S’ and the ‘K’) leading to a power-imbalance in the food chain, the farming sector having least power to secure prices and income (272, 273).	- Single parents and low-income households are especially vulnerable. - Domestic food production at acceptable prices is a challenge.	The share of household income to be spent on food if following FBDG would be 39% for people in the lowest decile group compared to 11% in the highest decile group in 2022 (171).	Swedish children’s food environment is dominated by fast food, snacks, and sweets as the majority of advertisements are about highly processed, unhealthy foods (274). Fast food, snacks, and sweets were easily available in shops nearby schools (275).
#16: Avoid adverse gender-related impacts, especially with regard to time and allocation.				
Cross country: The Nordics have come far in gender equality, but there are still many differences in various fields. According to the Global Gender Gap Report, Iceland remains the only economy to have closed more than 90% of its gender gap. Other Nordic countries such as Finland (86%, 2nd), Norway (84.5%, 3rd), and Sweden (82.2%, 5th) feature among the top 5 (276), while Demark comes on the 32nd place.				
Men have on average a higher energy intake than women, and a much higher intake of meat and meat products than women, but not higher intake of fruits and vegetables (277). However, when adjusted for energy intake there is no difference between men and women in the GHG emissions per MJ (278).	Agricultural labour force is dominated by men. Most women have work outside home but are still having more responsibility for grocery shopping, meal planning and cooking (45). Long family leaves affect women’s pension levels and career trajectories. A substantial disparity in the healthiness and sustainability of food consumption between genders (270–272).	According to the most recent study women are closer to the recommended FBDG than men (279).	Young men pull the average meat and dairy consumption up (280).Young women (18–24 years old) are much more likely to eat a vegetarian dinner than men (266).	Young men consume more meat than young women (267). Newer data confirms a difference in attitude where more men say they prefer to eat meat, while the consumer group ‘flexitarian’ is dominated by women (281).
**Opportunities**				
Principle # 9: Maintain greenhouse gas (GHG) emissions, water, and **c)** land use, nitrogen and phosphorus application, and chemical pollution within set targets (interpreted as targets within a country).				
Cross country: All five countries have high ambitions for GHG reductions, less water and land use changes, more careful use of N and P and increased production of organic foods to reduce pesticide use and improve soil health. All five countries also rank avoidance of peatland conversion high on the agenda.				
- Technological measures on way, for example, feed additives reducing methane emissions from cows, better biogas handling, etc. - Dietary shifts with less meat and dairy - Cleaning programs are successful - Rewetting of peatlands on way - Gradual replacement of synthetic pesticides in agriculture by integrated pest management (IPM) and organic farming (e.g. mechanical pest control)	- Incentives to abandon cultivation of peatlands (282) and implementation of high nature-value farming systems (104) - Increased consumption of domestically caught fish (283). - Increased production and consumption of legumes (284). - Free school meals and sustainability criteria in public procurement (52). - High self-sufficiency rate (see Table 1) allows better management of environmental impacts through domestic policy (285).	- Balanced grazing strategies, restoration of drained peatlands. - Increasing and diversifying production of vegetables. - Geothermal heat keeps energy prices relatively low. - Changed subsidisation scheme towards vegetable and fruit production	- (Flexible) ban on peatland conversion, potential changes in production (reduced animal and feed, increased plant-based food), changing subsidies, import tax, procurement agreement for plant foods, greenhouses (hydro-energy) – but challenge for latter: up front investments! - Stronger regulations, commitments to protection (EU, IPBES) and increased soil health and (various) carbon capture (EU, National climate targets) - Nitrate directive (max. nitrate load), crop rotations with legumes - Crop rotations to avoid pests/diseases - Stronger regulations, technological solutions (drones, monitoring, crop/time specific applications)	- Fiscal measures or other policy for the reduction of CO_2_, fertilizers and pesticides. - Changes in consumption patterns with more plant-based and low impact blue foods - Increased focus on growing and processing for increased value products of for example legumes - Support for rewetting peatlands (286) - Methods for more specific nitrogen application may be useful (287) Work within the EU on chemical pollution emphasizing producer responsibility (288).
Principle # 10: Preserve biodiversity, including that of crops, livestock, forest-derived foods, and aquatic genetic resources, and avoid overfishing and overhunting.				
Cross country: All five countries ratified the Convention on Biological Diversity in 1994, agreed to implement the Aichi biodiversity targets in 2010 (289) and signed the Montreal Kunming agreement in 2022 (90). All five countries have a knowledge gap in terms of documenting the food production’s impact on biodiversity both on land and at sea but have resources to fill this gap.				
Rewetting drained peatlands, increase percentage of protected land, rewilding (290). More protected land must be prioritized. Biodiversity impact abroad from the Danish dietary intake should be paid more attention in future guidelines.	Over 85–90% of the environmental impact of Finnish food consumption in terms of reducing global species richness is associated with imported products (247, 248). Thus, the focus should be on these imports. Decreased consumption of the foods with the largest global biodiversity impact in the Finnish diet: for example, poultry, imported aged cheese, pork and beef, and coffee (291). Advancement of novel agricultural methods, the upkeep and the establishment of new environments that promote biodiversity (291).	- Fishing strategy to be followed by law (292). Measuring fish stocks to avoid endangering (292). - Changing of eating habits to preserve and boost biodiversity both abroad (imported foods) and within Iceland is an opportunity which could support health and diminish land erosion - Indications for opportunities for increased vegetation and biodiversity is the fact that 65% of the country was covered by vegetation some centuries ago (293) and lowering consumption of sheep meat (294). - Continuous work on sustainability of fish stocks, preserving fish stocks and species to prevent overfishing and support biodiversity	- Biodiversity can be increased through implementation of flowerbeds along crop fields, crop rotations, and intercropping. - Reduced pesticide/chemical use - Increased outfield grazing but avoiding overgrazing and reducing total numbers of ruminants to not increase GHG). - Improved catch methods (fisheries), solutions to catch precipitation from fish farms	Development of new agricultural methods, maintenance, renovation of land and creation of new biodiversity rich environments are needed, along with monitoring of progress to reach the set objectives for biodiversity in agricultural landscapes (102).
Principle #11: Minimize the use of **a)** antibiotics and **b**) hormones in food production.				
Cross country: All five Nordic countries are in the forefront in low use of antibiotics, Norway, Iceland, and Sweden ranking lowest in Europe. The achievement is the result of good collaboration between primary industries, authorities, and research. Hormones in meat production is forbidden. The situation may be used as an opportunity to inspire other countries.				
Principle #12: Minimize the use of plastics and derivatives in food packaging.				
Cross country: 169 countries have recently agreed on an action plan to reach the plastic targets (295). The European Green Deal agreement 2022–2027 aims to reduce the consumption of single-use plastic cups and certain food packages (129). In addition, the individual Nordic countries have their own ambitions, for example, Iceland, with regulations and actions to reduce the usage of plastics (296). Opportunities include reusable packaging options, get rid of unnecessary packaging, limit overpackaging, and provide clear labels to support correct recycling.				
Principle #13: Reduce food loss and waste.				
Cross country: Table 1 gives numbers for gross household waste per country. An opportunity taken by all five countries is their monitoring and reporting of food waste, enabled by better collaboration throughout the food chain. This enables targeted actions (297). Sweden reports a decreasing trend for food waste in households (136). Opportunities include smarter public and private procurement (buy what you need), smaller plates when serving, food waste agreements, product date marking ‘best before not bad after …’				
Principle #14: Are built on and respect **a**) local culture, culinary practices, knowledge, and consumption patterns, and **b)** values on the way food is sourced, produced, and consumed.				
Cross country: There is an increased interest in healthy, sustainable, and locally produced foods in all five countries. This is reflected in a high interest in more plant-based, organic, and locally produced food in public meals, especially among the young (264, 298). Politicians may see this as an opportunity to increase self-sufficiency. The trends increase the opportunity for national resource-based plant-based food innovation and general awareness raising. System innovation initiatives on school meals with local prototypes are underway. There is also experimenting with locally sourced food, education and student involvement (299), for example, unconventional seafood (300). People generally rank domestic origin as one of top-quality aspects for meat as well as plant-based foods (281).				
#15: Are accessible and desirable.				
Cross country: As shown in Table 4, four of the five Nordic countries rank among the top 15 countries on the Global Food Security Index (GFSI), Finland being #1, in spite of the countries having very varying degrees of self-sufficiency (see Table 1). Reduce dependency on imported fodder ingredients would increase food security, thus ensuring accessibility also in more extreme situations. Both Finland and Sweden serve free school lunches to pre-primary, basic and upper secondary education pupils, an opportunity for the rest of the Nordics to make healthy and sustainable meals available for children from all socioeconomic groups (301, 302). A variety of plant-based foods should be accessible and affordable regardless of income (182), but market access is often a limiting factor for small-scale farmers. There are promising initiatives to improve social and physical environment to better support sustainable and healthy food choices (e.g. (303)). The Norwegian Government has recently agreed to follow up on two issues: 1) to legislate a ban on the marketing of unhealthy food and drink aimed at children and young people under the age of 18 AND 2) to put forward a proposal to introduce a 16-year age limit for the purchase and sale of energy drinks (304).				
#16: Avoid adverse gender-related impacts, especially with regard to time and allocation.				
Cross country: Not stigmatizing use of convenience foods (as this may affect women more than men) but rather work for healthier convenience foods available? This could be a subject for innovation in Nordic countries. Family leave policies that aim to an equal distribution of child-care related leaves between spouses could be supported and further developed.				

For all tables: Examples of domestic challenges and opportunities when incorporating sustainability into food-based dietary guidelines in the Nordics: Green = environmental aspects, yellow = sociocultural aspects.

To follow up on UN’s SDG12.3, indicators have been developed. To support food waste reporting, large efforts have been made to align the EU-reporting and the reporting towards SDG12.3 as much as possible. Sweden and Denmark link their reporting closely to the Waste Framework Directive, while Finland and Norway base their data collection mostly on voluntary reporting. Norway and Finland report on a detailed level and estimate impact like costs and GHG emissions. The Icelandic government in 2021 initiated a plan to minimize food loss and waste monitored by the Icelandic Environmental Agency (141). All Nordic countries have detailed data that fulfil the requirements set by the purpose of food waste monitoring program (142).

The Natural Resources Institute Finland (Luke) has built a national food waste monitoring system through a dedicated project (143). The project has developed tools for monitoring and reporting on food losses and waste with the aim to identify the most efficient measurement methods for each stage of the food chain.

However, more than anything, a transition toward circular food systems is needed (88). Post-consumer food waste can be safe and nutritious for pigs when treated properly (144), and pre-consumer, plant-based food waste can also be fed to ruminants (145). Replacing food-competing feedstuff with food waste could save up to 8.8 million tons of human-edible grains in the European Union (146), in addition to estimates of 14.7–18.6 million tons on the replacement potential of cereals with by-products and crop residues in Europe (88).

A relatively low proportion of household income is used for food in the Nordics (Table 1), which reduces economic incentives for lowering food losses at household level. Over-consumption leads to environmental impacts with no nutritional or culinary benefits. A more circular approach to food production and waste management can be part of the solution for more sustainable food production systems, like the one initiated by the Icelandic government in 2021 (141).

Use of all cuts and organs of the animal and seafood is important for efficient use of resources. Traditional knowledge and recipes of non-filet parts of animals would be useful for avoiding wasting edible parts of animals. Using the whole animal and seafood implies developing and producing more processed meat and seafood products, which is in conflict with the health advice to reduce the intake of processed meat. In a paper by Ascheman-Witzel et al. (147), examples are provided of upcycling food waste in the food industry and thus how to generate additional revenue for the industry while lowering environmental impacts.

A Nordic report on food loss and waste states the following: ‘Halving food waste by 2030 calls for radical changes in the food chain. These radical changes require four dimensions: technology push, societal pull, market pull, and regulatory push. Based on these four dimensions, measures to reduce food waste were classified into four topics: Policy instruments, changing social norms, nudging and changing practices, and intelligent technology and new products and business models” (142). To halve food waste, key actors from all steps in the food chain need to collaborate to agree upon the methods and solutions.

## Sociocultural aspects

### Principle #14 Are built on and respect local culture, culinary practices, knowledge and consumption patterns, and values on the way food is sourced, produced, and consumed

Respect for local culture may be regarded as cultural acceptability, meaning that recommendations and advice should not diverge unnecessarily from established dietary habits and production patterns (which, however, may not be very good sustainability-wise today), including their social contexts (148). However, what constitutes ‘established dietary habits’ is a moving target: The inter-connectivity of the global food system supply chains in most high-income countries has led to a shift from more traditional diets composed of a limited set of staples toward more diversified diets that are higher in energy and macronutrients (149, 150). This dietary change is not least visible in the Nordics, where all five countries have become increasingly embedded in the global food market (21, 37). From the producers’ perspective, in a global food system, changing consumption patterns may impact negatively on other countries culture, for example, through changing production patterns that re-shape landscapes and peoples’ access to food.

Accordingly, the food basket in all Nordic countries has changed formidably from the 1950s till today. It contains more fruit, vegetables, and meat, but less fish, milk, and potatoes (38, 151–154). We now buy fruit and vegetables all year round, the diet has become more varied and is generally more similar to the diet in other affluent societies. Many people eat healthier, but most people eat far less fruit and vegetables than recommended (5). Our modern diet also implies a far greater consumption of highly processed foods with a high content of salt, added sugar, and/or saturated fat. Many such products are cheap and lead to a high consumption of soft drinks, biscuits and snacks, although purchases of sugar-containing soft drinks have declined over the last couple of decades in some countries (38, 155, 156), but increased, for example, in Denmark the last decade (157). On the other hand, artificially sweetened soft-drink and energy-drink sales have surged in countries like Norway and surpassed the sales of sugar-containing soft drinks severalfold (158).^5^

These poor diets are a leading risk factor for human health across the region, responsible for 40–48% of deaths from cardiovascular disease and 25–28% of deaths from diabetes (159). Overconsumption of energy-dense foods contributes to half of the adult population and one in seven children being overweight or obese (160, 161). Excess consumption of processed meats is also a risk factor for cardiovascular diseases and colorectal cancer (162, 163)

Nordic food consumption may cause high pressure on biodiversity in sensitive ecosystems in other parts of the world through high food imports. One aspect of valuing the way food is sourced, produced, and consumed is providing help in mitigation of the problems caused (105). Many developing countries depend on the export of, for example, coffee, tea, tropical fruits, and vegetables for their economy (164), raising ethical challenges of all diets (165). Utilization of feed resources for aquaculture and animal farming, in general, also raises ethical questions about efficient and equitably use of global food resources (166).

A challenge overall is that food culture along with heritage of landscapes are not properly accounted for, one example being the Sami food culture and traditions of reindeer keeping. The economic value of an efficient food production is often prioritized over sociocultural aspects, and the neglect of such local cultural aspects may also increase polarization when food policy is discussed.

### Principle #15 Are accessible and desirable

Food security may be assessed by the Global Food Security Index (GFSI) that evaluates food security across four key pillars: affordability, availability, quality and safety, and sustainability, and adaptation (167). Among the 113 countries that are ranked, four of the five Nordic countries are included.

Eight of the top 10 performing countries in 2022 are in Europe, three of them are from the Nordics Incert (Table 4).

**Table 4:** Global Food Security Index 2022 (167) must be incerted here, the reference. max score being 100 in four Nordic countries*

Parameter	DK	FI	NO	SE
Overall score/rank	77.8/14	83.7/1	80.5/3	79.1/7
Affordability/rank	92.1/6	91.9/8	87.2/28	91.9/7
Availability/rank	63.2/39	70.5/16	60.4/51	68.3/21
Quality and Safety/rank	89.1/2	88.4/5	86.8/8	85.0/11
Sustainability adaption/rank	63.8/24	82.6/2	87.4/1	68.3/14

The first value is the score and the second is the rank.

* Iceland: no data. Affordability: Measures the ability of consumers to purchase food, their vulnerability to price shocks, and the presence of programmes and policies to support consumers when shocks occur. Availability: Measures agricultural production and on-farm capabilities, the risk of supply disruption, national capacity to disseminate food and research efforts to expand agricultural output. Quality and safety: Measure the variety and nutritional quality of average diets, as well as the safety of food. Sustainability and adaptation: Assess a country’s exposure to the impacts of climate change; its susceptibility to natural resource risks; and how the country is adapting to these risks.

Although the Nordics overall get high rankings, the scores in the 2022 GFSI reflect a fragile global food system that is under immense pressure and increasingly risks very bad outcomes. Globally, food prices and hunger have recently been hitting record highs, while affordability is plummeting as shocks like the COVID-19 pandemic, armed conflict, and climate change compound systemic stresses. These stresses and shocks pose risks that could get worse as threats to food security become the new normal (167). Special support may be needed for the most vulnerable population groups whose food security has been permanently weakened (168, 169).

Sadly, to many, a healthy and sustainable diet is out of economic reach (19, 170). In Norway, a recent investigation showed that the share of household income to be spent on food if following FBDG would be 39% for people in the lowest decile income group compared to 11% in the highest decile group (171). However, with knowledge and skills, some seem to manage on a low budget (172).

An important dimension of national food policies is to balance self-sufficiency in, at least, what could be called basic foods for national food security, with market driven networking in the global food markets. Global market is less, while domestic food chains are more in reach of national policies. The self-sufficiency and food security dimensions are not explicitly covered in principle #15, so this is covered more in the discussion.

### Principle #16 Avoid adverse gender-related impacts, especially with regard to time and allocation

The Nordic countries have a long history of cooperating and sharing knowledge on gender equality. The cooperation is driven by a shared vision of a gender equitable Nordic region with equal opportunities, power, rights, and obligations for all genders (173). Although the Nordics have come far in gender-related issues, there are still striking gender imbalances within many fields. As illustrated by data from Finland, in agriculture, the labour force is male-dominated, while in the food industry, gender proportions are evenly distributed (174). On average, women in Finland spend more time tending to domestic duties and less time on paid work than men. In a Swedish study, women’s health was negatively affected in households with uneven sharing of household duties (175). Even in the Nordic countries where most women are working outside home, the traditional role of women as caregivers for the family remains, illustrated by having more responsibility for grocery shopping, meal planning, and cooking.

There are several examples of gender differences in the environmental impacts of diets (5). In high-income countries such as the Nordics, women and individuals with higher socioeconomic position (higher education or higher income) tend to consume more vegetables and less red meat than men and those with lower socioeconomic position (155, 176–179). There may also be disparity between the diets of nonbinary and binary genders, which should be studied (180). The unhealthiest food habits are found in young adults, for both men and women (181). One of the challenges in affecting change lies in not exacerbating the present gender differences in food consumption.

In Finland, men start the dietary transition toward environmental sustainability far behind women, as illustrated by (155, 182). In terms of GHG, men’s diets at their lowest levels of associated emissions are higher than those of women at their highest levels (183). Part of the larger dietary climate impact of men is explained by the larger energy requirement for men compared to women (5, 184).

The dietary climate impact varied considerably between individuals in a cohort of 59–95-year-olds in Sweden; women and older individuals had the lowest climate impact (185). The climate impact was driven by the consumption of animal-based foods as these foods have the highest product-based impact. Dairy had the largest climate impact for women and red meat had the largest climate impact for men in this population group (185). The impact differences were larger for certain food groups with a striking example being that the alcohol intake among men in Sweden generated about 90% more GHG emissions than the alcohol intake by women (185).

Some indications of adverse health effects were observed in men with diets that had lower climate impact but also with less nutrient density than a reference group, suggesting that more climate-sustainable diets are not necessarily synonymous with healthier diets (186). It is important to consider nutrient quality and not to assume similar effects of dietary changes for men and for women as there are gender and age-related differences in disease patterns.

## Some Nordic food system related aspects of sustainability

The FAO/WHO principles #9 – #16 constitute necessary, but insufficient conditions for generally adequate food system sustainability. Because the sustainability field is so large, it is not practically possible to go into all details that are significant in the Nordic context, but we here provide a short coverage of important issues related to, but not directly covered by the framework of the FAO/WHO principles.

One issue that is highly debated in the public discourse on food and sustainability is the role of self-sufficiency in food security. Food security is increasingly vulnerable at the global scale, while being a fundamental aspect of sustainability, and it involves global, regional, and national food systems. To what degree, and at what social-geographical scales does self-sufficiency in food production improve food security? It is particularly important to put food self-sufficiency in its proper perspective:

*‘Food self-sufficiency is often presented as an extreme and isolationist concept by its critics, who see it as inefficient and trade distorting. In practice, however, many countries seeking to improve their food self-sufficiency do so in the context of international trade. The aim is not to produce 100 percent of their food on domestic soil, but rather to increase domestic capacity to produce food, even if the country engages in food imports and exports. The narrow focus of the debate fosters an “either, or” approach that downplays the real concerns of many countries regarding their domestic food production and its implications for their food security, political stability, and economic development’.* (34)

Globalizing food appears as consolidation of actors, as decrease in the diversity of production practices, and homogenization of food cultures (187, 188). Production of the major commodities traded globally is concentrated to a few regions in the world. Almost a quarter of food production is traded internationally (189), This has increased supply diversity, and the market serves as a buffer and backup against disruptions in local systems (21, 190). On the other hand, increased dependencies on global trade of inputs for production for feed and food products have created new types of vulnerabilities, such as, for example, experienced during the COVID-19 pandemic (191).

The food-industrial business logic for the global market favors large-scale production and strong specialization to reduce marginal costs and gain comparative advantage. This has supported the trend towards more uniform production structures in agroecological regions and contributed to loss of diversity at several levels (192). This has led to increasing displacement of rural smallholder farmers from their livelihoods, and over past 30 years, loss of around 200 million jobs in farming, migration to urban peripheries, and unemployment. This appears as negligence of the significant contribution to food security these local production systems make (193).

Reductions in regional and national production diversities (194) can be counteracted by existing parallel processes of diversification of food production within industrialized countries (195, 196). EASAC (196, chapter 2.2) concludes that ‘the recent discourse on localizing food for dietary diversity and food system resilience is likely based on yet incomplete understanding of the dynamics of the food and production systems. However, what is clear is that the trend of uniformity of diets towards a “global diet” drives export-oriented agribusinesses towards simplification, monocultures, and homogenization of agricultural landscapes and farming systems’ (197).

### The interplay between local, regional, and global food systems

Historically, the Nordic countries have had relatively open and strong economies, and extensive trade of foods and other agricultural products has been both natural and important. Today, the Nordic region relies on inputs from around the world to keep the food system going – whether that be imported food, feed, fertilisers, or energy to enable farms; foreign labour for harvesting; or importing of knowledge and skills from around the world. There is no way to clearly separate the global food system from the regional food system. However, large dependency on food imports not balanced by a corresponding (on a relevant scale) exports is increasingly problematic by sustainability and resilience perspectives.

For food security and optimal resource use, making the most out of the opportunities of the local food systems is essential, but this must happen subject to environmental, social, health, and food system-related constraints. Power, control, and decision processes are also important aspects of general sustainability. Resilience will often depend on good solutions to such issues, for example, empowering stakeholders and developing good strategies to take care of vulnerabilities.

The solutions easiest to maintain will often be associated with food systems with transactions at the local and regional level dominating, and the global food system providing products important for the functioning of the regional and local systems. This includes ‘product backup’, like safeguarding against food shortages. Such global backup will be more efficient the better the safeguarding at the local and regional level is (198).

The traditional local Nordic food systems have, generally, been quite resilient, with a large degree of seasonality and flexibility, adapting to differences in supply, for example, by varying the amount or form of meat used in dishes, at times substituting fish or legumes for meat and dairy. With constant access to the global food system, local produce can be used even more efficiently, utilizing imported fruit, vegetables, and spices to help create attractive dishes based mostly on local vegetables and other produce. In the same way, modest amounts of imported nuts and legumes may contribute to increased use of locally grown cereals and domestic dairy products.

On the feed side, high-quality ingredients, like soy, are used for improving the quality of feed concentrates based mainly on Nordic grains and oilseed residues. Using some imported soy allows for larger use of local ingredients of lesser quality (199).

If priority is given to balancing the flows in the food systems, net imports may end up being quite low. Regional food systems, like the Nordic/Baltic, can play an important role in the interface between local systems and the global food system.

The case of Denmark demonstrates that very good integration with the global food system in no way by itself provides sustainability of local consumption, and this aspect must be considered separately. Current domestic meat supply in Denmark is more than twice the global average (Table 5), and, as noted above, is for a considerable part based on feed resources that are in large and increasing global demand for human food, the supply of which is increasingly insecure (201). Thus, the very high Danish consumption is, in principle, in conflict with SDG 2.

**Table 5:** Net supply of red meats and poultry per capita, grams per week, 2020. From FAOSTAT Food Balance Sheets (200), Nordics and the world

Country/area	Bovine	Pig	Mutton/Goat	Red meat	Poultry	Total
Denmark	337	723	13	1,073	324	1,397
Finland	236	385	12	633	348	981
Iceland	226	302	301	829	415	1,244
Norway	247	328	76	651	261	912
Sweden	294	394	18	706	267	973
Nordics	281	448	26	755	297	1,052
World	121	182	35	338	221	559

In addition to improved food security in a general sense, better control of the global environmental impact is desirable. For the Nordic counties, optimizing the use of local resources with respect to total human food production and environmental footprint, combined with better integration with global food systems, may reduce the current global environmental impact and related ‘social footprint’ abroad. This may be considered a part of responsible integration with the global food system, applying indicators of impact and footprint as, for example, reflected in the spill-over index (10).

### Local production

As noted above, local production and a certain degree of self-sufficiency are important for food security and optimal resource use. In addition, economic organization and collective actions (political and otherwise) may also be important for social and economic sustainability.

For several decades, economics of scale and international competition tended to reduce the general importance of local production. In the Nordics, diversification of diets contributed to reducing the scope of local production. This has largely been a consequence of better availability and reduced prices of agricultural commodities. For example, in Norway about 2/3 of the agricultural area is currently used almost exclusively for grass, while historically, the production on these areas has been more diversified. The specialization happened mostly because of regional political concerns, economic efficiency, and development of farming methods. New species and cultivars along with climate change may, especially under favorable market conditions and supported by strong governmental incentives, result in renewed diversification. However, market access, generally, and the actions of the dominating food retail chains, particularly, may be more decisive (202).

A food system with a large supply from diversified local production will, generally, be more resilient than a system with fewer production sources. But, as demonstrated by the 2018 drought in parts of the Nordics, local supply may become more vulnerable over time under climate change with larger variability and more weather extremes. It is, therefore, very important that the food system can accommodate the perturbations that may occur in varying environmental and market conditions. Diversified production is one key factor here.

If local production does not meet strict environmental and social criteria, it is not sustainable and should not be considered part of sustainability driven dietary regimes. In the Nordics, influence on environment, climate, biodiversity, and soil health are some of the criteria that must be checked and monitored. Generally, compliance with the Farm to Fork criteria will represent a major step toward environmental sustainability (46).

### Social sustainability and resilience

Affordability and accessibility, as described in Principle #15 above, are important aspects of social sustainability. They should be considered within the more general framework of social sustainability. For example, local special products may not be very affordable, and in some cases not even very accessible, but they may still have very high sustainability scores in most respects and be important elements in sustainable diets.

When applying a food system perspective, it is important that all people involved with the food system, not only the consumers, are protected from poverty and have at least their basic needs fulfilled. This may be hard or even impossible to achieve if the food systems are large, complex, and nontransparent, in particular when the system dynamics are dominated by profit concerns and strong power relations. This may be an important argument for higher emphasis on local or regional food systems if they can provide better social control than more globalized systems.

When social sustainability is taken into consideration, the global food system will always be implicitly involved, as there is no way to set absolute limits for social concerns. For example, if social sustainability is invoked to give local food production higher priority, assessments must include eventual dependency of and effects on the global food system.

Resilience is a complex concept and in several aspects dependent on policy. A resilient system, by definition, maintains its functionality within a wide range of perturbations and shocks. For example, according to the EU commission, a resilient food production system is not only environmentally sustainable but also ensures sufficient income for all farmers, in particular for small- and medium-sized farms vulnerable to income volatility. In this context, economic resilience may come in conflict with environmental resilience. For example, practices that are economically sound and locally environmentally acceptable may violate planetary boundaries. Resilient production is attained through a policy framework and an effective set of policy instruments and mechanisms, together with reduced dependence on fossil fuels as well as balanced imported inputs to optimize system performance and robustness. If production is reduced, making food supplies scarce, ordinary export may be put on hold, but exchange of products will often still be advantageous for the trading parts (203).

### Economic sustainability

Economic aspects may be the most difficult aspect of sustainability to handle because it normally involves combining economic growth with social and environmental sustainability, and the handling of wealth and burden distribution issues connected with growth is, generally, controversial. Increasing inequalities and growing poverty among groups, including farmers, demonstrate that the policies applied may be considered inadequate by many. Yet it is an essential feature of food systems and must always be considered. This is very important to handle because so much of the social fabric is strongly influenced by it (204). If politics and market conditions are aligned so it is economically favorable to develop in sustainable directions, the social tensions may be reduced.

### Nordic perspectives on livestock

Even though the Nordic food systems are under pressure to become more plant based, and even though an exclusively Nordic food system could provide most of the alimentaries needed for even a vegan diet (205), it must be assumed that livestock for the foreseeable future will represent a main element in Nordic food systems, with herd sizes and production patterns and volumes that both make efficient use of resources and comply with constraints on emissions. When ruminant herd sizes are large, optimal use of grass resources may not be possible because of limited possibilities for grazing. For feed supply, there are currently essential contributions from food industry waste and side streams like cuts from sugar production and press cakes from rapeseed processing (114). There will also always be a part of crops that are best or only suited as animal fodder, but attempting to precisely quantify this is a futile exercise. For example, using high-yielding grain varieties will usually result in a considerable proportion of feed-grade crops under unfavorable weather conditions, but overall, this may be an optimal strategy. If maximizing the proportion of crops suitable for human consumption had been the main goal, the optimal strategy could, however, have been quite different (88, 206).

From a sustainability perspective, Nordic meat consumption is closely related to regional meat production, but the role of the Nordics in the global food system must also be considered. As the Nordics have several comparative advantages in livestock production (including not only relative advantage in grassland production but also ample renewable water resources) relative to the world average, the local and regional food systems should be considered within a global context. This includes the feed issues, for example, how food industry waste streams are best used from a sustainability perspective. This is another example of the interaction between self-sufficiency and global food system concerns.

To varying degrees, imports of feed ingredients are instrumental in aquaculture, meat, and dairy production in all the Nordic countries. This represents one of the least sustainable aspects of Nordic food production and consumption. For example, rather than the 20–30% feed concentrate often used in organic dairy farming, milk production may be based on about 40%, or even more, feed concentrate (207, 208). This has reduced the GHG emissions per unit milk produced, but not total emissions from cattle, because the high demand for beef leads to increased use of suckler cows, exemplified by the Norwegian situation (209). Consequently, a stronger emphasis on self-sufficiency and use of local resources would in most cases reduce the dairy or meat output in the short term, but innovations may over time increase this output.

The options of a more self-sufficient Nordic food system with lower output of meat and dairy than today was, for example, addressed in a Nordic vision of a sustainable diet (210). On the other hand, as pointed out above in this review, a sustainable global role of the Nordic countries could be to produce dairy based meat and milk products to global markets, hence increasing production while domestic consumption is adjusted to dietary recommendations (74, 211).

### Meat consumption

Because of the large grass resources, a somewhat higher red meat supply than the average global of ca 340 g/week, for example, 350–390 g/week, is also to be expected with the normal local upweighting in food systems; foods produced on domestic resources tend to be used more. The current average Nordic red meat supply is, however, 2–3 times the global (Table 5), and for a considerable extent based on imported feed ingredients. At the same time, utilization of local grass resources is suboptimal. For dairy production, the situation is similar, with almost 40% average feed concentrate share in milk production in the Nordics. Seen in isolation, a large part of feed concentrate for dairy and beef production is locally produced and its use seemingly not stressing the global food system. But when the net self-sufficiency is, for example, 40% as measured by the method in general use in Norway (see abbreviation list), the totality of domestic food production and consumption stresses the global food system. This can be amended in at least two ways: Using local resources (e.g. grass) better, and reducing consumption, exporting the resulting surplus of meat and dairy. Sheep and goats may be part of flexible solutions to such challenges, utilizing marginal feed resources. Nordic production of such meat per capita is only 8% of average global red meat supply, see Table 5. Thus, if prioritized, it could be increased several-fold without stressing domestic feed supply and providing eventual surplus globally fits with local food traditions in several regions.

### Nordic perspectives on seafood

The Nordic region is a large supplier of seafood, which has often been considered as inherently problematic from a sustainability perspective because of widespread problems with over-fishing and environmental problems connected with aquaculture practices, and land use changes connected with feed production. When control mechanisms are in place securing adequate animal health and welfare, seafood can represent a good and sustainable alternative food source with low GHG emissions, especially if compared to some red meat alternatives (58). The potential for lower trophic aquaculture is yet to be developed in the Nordic countries. The seafood industry is, however, very diverse with respect to both environmental performance and nutrition. Generally, farmed fish does rather well in feed-feed competition with livestock, making very efficient use of the feed ingredients used.

There are several environmental issues involved, among them animal welfare issues, bottom trawling for fodder fish and sea-floor ecosystem damage from fish farming (58, 212).

### Nordic perspectives on plant-based diets

With the interest in plant-based diets surging, particularly among younger people in the Nordic countries, it is important to note that such diets, with some nutritional knowledge, can cover all nutrient needs, particularly if some food supplements are used. Studies of vegetarians suggest that such diets may reduce the risk of several NCDs (213–216). However, following a plant-based diet is no guarantee against imbalanced nutrition, particularly if access to a variety of plant-based foods, including fortified ones, is limited. This may in part explain why in the Nordic countries, some studies have concluded that adherence to plant-based diets may also be associated with health risks (217–219). This highlights the need for specific FBDG as well as adapting national food policies and local food production to this growing group of consumers. Plant-based foods are necessary parts of viable sustainability pathways. It is important to ensure a broad and thorough public information about the need for supplementing food with essential nutrients in the case of total avoidance of all animal-based foods. To increase availability of accessible and nutritious plant-based foods, there is potential for increased production and processing of, for example, vegetables and legumes in the Nordic countries (220, 221).

### Net Zero and agricultural GHG emissions

Under a standard interpretation of net-zero emission, non-CO_2_ emissions like agricultural methane and nitrous oxide must be reduced, but eliminating these will be difficult if not impossible. In the Nordic agricultural context, CO_2_ emissions from Nordic organic soils contribute about as much as nitrous oxide and methane (Table 2), and emissions associated with land use for agricultural production are therefore also a considerable concern. Food production practices must aim at reducing all GHG emissions, but some of this work may take a long time. It is possible to drastically reduce enteric methane production in ruminants, and research is ongoing to find, combine, and compare efficient, immediately applicable methods (15). It is also possible to reduce the nitrous oxide emissions (222). Very efficient solutions may be developed over time, but there are no quick fixes. Highest immediate priority must in any case be given to eliminate emissions that cause further global warming. When methane emissions are gradually reduced, they contribute to sustaining existing global warming, but as their relative forcing decreases, they do not directly cause further warming (30).

While Net Zero is a necessary condition to be reached as soon as possible, the path to get there is also of great importance (223). For all GHG emissions, the faster they are reduced, the better. Reducing agricultural methane emissions rapidly would result in a reduced warming effect, but in this context it is important also to be aware of the large and increasing methane emissions from natural Nordic wetlands (224). There has been an 165% increase in atmospheric methane relative to pre-industrial levels, while the corresponding increase in nitrous oxide has only been ca 25% (225).

## Discussion

Food system sustainability is an extremely wide and diverse field, and assessments may be expected to vary over time, with changing methodology, urgencies, focuses, and priorities. Working for food production and consumption, including the whole diet to become more sustainable, means working for a continuous improvement that enables development of resilient food systems and food security, efficient, and robust value chains, strengthens public health, improves the food system locally and globally, and reduces food’s negative environmental impact. Here, we have identified many cross-scale and intertwined challenges that are mostly common but partly varying between Nordic countries. We have tried to take a systemic approach to ways of handling them, and shown that actual paths forward will have to involve most stakeholder groups or food system actors. We believe such an approach will lead to a more robust path towards a sustainable food system.

Traditionally, the environment, in particular GHG emissions contributing to climate change, have had a prominent place in sustainability considerations. In the Nordics, there is a conflict between minimization of methane emissions from ruminants and utilization of grass for feed, which is central to agroecology. This is just one example of the general principle that to proceed efficiently with sustainability criteria, the overall consequences of any proposed changes need to be carefully assessed. Such an assessment must be done with both qualitative and quantitative methods to acknowledge the social-biophysical nature of the system, thus providing the foundation for sound political decisions (3).

As always when economic stakes are high, interest groups may perceive a threat from sustainability considerations and act to provoke a polarised social debate about sustainable diets. The aim of such polarised debates will often be obstruction rather than clarification. To counteract obstruction efficiently, the current state of knowledge must be presented as precisely as possible, and the necessity to sometimes act on incomplete knowledge must be clearly explained. For example, biodiversity may never be completely understood in all its aspects, but using that as an argument for doing nothing or too little is a recipe for further biodiversity loss. The same holds for using estimates of GHG emissions that often vary depending on methodology, accounting rules and underlying assumptions. The best available estimates may be useful for guiding action, even with large error margins. In presenting the current knowledge precisely, it is also important to become explicit about the existing conflicts of goals and pathways to these in aiming to sustainable food systems. These are the very issues where policy decisions are difficult.

### Increased emphasis on food security

Global demand and variability in crop production may both be expected to increase, creating a more volatile global food and feed market (167). High reliance on a few supply chains on the global supply market for agricultural and food industry, and on the food market, have turned out to be vulnerable to crises, as recently shown by the war in Ukraine, and the COVID pandemic, and even to minor unexpected events, such as exemplified by a few days accidental blockage of the Suez Channel. Hence, the quest for resilience in food security needs to reconsider dependencies on few and long supply chains for food and resources needed for food production. Warmer climate may ease the transitions to a higher degree of Nordic self-sufficiency for growing certain oilseeds and protein-rich plants, but more extreme weather patterns may reduce those benefits and introduce new vulnerabilities, see, for example, (19, 75).

When integrating self-sufficiency and sustainability, one needs to consider what local and regional transition paths exist or can be made available. Policies to increase national supplies of grain and legumes for food, like in Finland, are a practical example of how to increase national food supply in synergy with the aim to achieve health and environmental goals through transition to more plant-based diets. At the same time, the Nordic countries have many relative advantages in livestock production, especially for grass-based dairy and meat production. If average use of feed concentrate to ruminants is reduced from the current high levels, and not substituted by new, inexpensive sources of protein and carbohydrates, the total number of animals must be reduced, proportionally to total feed supply. While methane emissions per unit produced may increase, total emissions will drop, and some more grass resources may be utilized without increasing total GHG emissions. As several approaches seem to work to improve control over enteric methane production, improved feeding regimes may over time allow for even higher resource utilization by ruminants.

Though their future contribution is uncertain, recent innovations in food production technologies (‘food frontiers’) may offer gains in ecological sustainability and global food security. A review of five frontiers is given by Glaros et al., including cellular agriculture, climate-driven northern agricultural expansion, controlled environment agriculture, entomophagy (insects), and seaweed and other low trophic aquaculture (226). In addition, animal feeds produced from forest by-products are being researched in the Nordics (227).

The main dietary modifications necessary are, generally, closely related to current vegetarian dietary diversification, which is mostly covered by nuts, fruits, legumes and vegetables, and an increased use of whole grain cereals. In response to this, a larger part of domestic grains could be directed to human consumption instead of feed, and more crop land could also be allocated to cultivation of other plant-based foods or restoration to forests and wetlands. With reduced meat and dairy consumption, net feed imports per capita to the Nordics will likely be reduced, while import of some plant-based foods, particularly nuts, oils, fruits, and legumes may increase, especially in the short term, but volume- or protein-wise, this will be outweighed by the reduced per capita feed import. Local production may also be supported by agricultural and trade policies. As an example of the large, unused Nordic potentials, the annual blueberry and lingonberry production in Norwegian forests alone has been estimated to cover the recommended 2-a-day of fruits for Norwegians (228). Nordic fruit yields are to a large degree constrained by production costs and market access, and much less from problems associated with production (202).

Globally, vegetables and fruits production each account for 4–5% of the agricultural area (42). In the Nordics, a substantial fraction of vegetables consumption is currently covered by local production, and at least technically, over time an increasing part of fruit and vegetables consumption could be covered by Nordic produce if measures to achieve this are given priority. When self-sufficiency is assessed on the basis of dietary energy contribution or land use, the import needed for dietary variation is for most diets less than 10% of energy supply or land use (38). However, a grave global problem is that to many, a healthy and sustainable diet is out of economic reach (169, 170). Thus, a natural part of policies for implementing food security and resilience would be to ensure that people can afford and implement a healthy diet.

## Conclusion

Geographical closeness, common values, well-functioning social welfare systems, and common ambitious goals for achieving sustainability, place the Nordic countries in a unique position to develop and implement sustainability-based policies for food production and consumption. Such policies should support optimal health as well as providing a basis for constructive roles for the Nordics in the global food system. There are numerous challenges but also many opportunities on the path to good compliance with the SDGs. Incentives to further development of the Nordic production systems should be continued in parallel with incentives to changes in the diet. Dietary guidelines need to include broader sustainability goals, not compromising human health but combining it with planetary health and sociocultural acceptability in a consistent way. This way, dietary guidelines can be used to advance both human health and wider sustainability goals.

## Footnotes

1 The spillover index describes a country’s negative socioeconomic and environmental spillovers, including through unsustainable trade and supply chains, that is, a country’s footprint abroad.

2 The core author team consisted of Helle Margrete Meltzer, Trond Arild Ydersbond, Ellen Trolle, Hanna Eneroth and Maijaliisa Erkkola.

3 Measured as flight km from Malmø, the southernmost town in Sweden, to Nordkapp, the northernmost point of Norway.

4 The European Soy Monitor data for Denmark has way too high consumption data for Denmark. When checked against soy use estimates, the error seems to come mainly from missing or too low figures for dairy export. Therefore, an estimate of ca 600,000 tons soy for dairy exports was subtracted from the figure to give a corrected estimate of approx. 550,000 tons for domestic use.

5 The scores are computed as weighted means of a set of indicators in each dimension. Indicator scores are normalized (min-max rescaling) and are scaled from 0 to 100, with 100 corresponding to the most favorable situation. The scores at the dimension level correspond to the weighted mean of underlying indicators. The overall GFSI score is a weighted average of the dimension scores.
